# Healthy lifestyle, daytime sleepiness, and gut microbiome composition are determinants of functional strength in humans: a cross-sectional study

**DOI:** 10.1038/s41598-025-02519-5

**Published:** 2025-05-19

**Authors:** Friederike Norkeweit, Kristina Schlicht, Nathalie Rohmann, Katharina Hartmann, Kathrin Türk, Ute Settgast, Dominik M. Schulte, Felix Gilbert, Tobias Demetrowitsch, Fynn Brix, Corinna Bang, Andre Franke, Karin Schwarz, Matthias Laudes, Corinna Geisler

**Affiliations:** 1 Institute of Diabetes and Clinical Metabolic Research, University Medical Center Schleswig-Holstein and Kiel University, 24105 Kiel, Germany; 2 Division of Endocrinology, Diabetes and Clinical Nutrition, Department of Internal Medicine I, University Medical Center Schleswig-Holstein, Campus Kiel, 24105 Kiel, Germany; 3 Institute of Clinical Molecular Biology (IKMB), Kiel University, 24118 Kiel, Germany; 4 Division of Food Technology, Institute of Human Nutrition and Food Science, Kiel University, 24105 Kiel, Germany

**Keywords:** Gut microbiota diversity, Microbiota interactions, Metabolomics, Strength, Activity, Cohort study, Epidemiology, Public health, Nutrition, Microbiome, Metabolomics

## Abstract

Age, metabolic inflammation, sleep patterns, lifestyle choices, and gut microbiome composition were investigated as factors influencing functional strength. The Northern German FoCus cohort subgroup (394 women, 233 men) was categorized into six groups based on weekly sports activity and handgrip strength (HGS) measurements. The analyses included anthropometric data, clinical biochemistry, medication, sleep duration, healthy lifestyle score (HLS), 16 S rRNA gut microbiota, serum and urine metabolomics, bile acids, and an adapted dietary inflammatory index (ADII) score. Associations were found between age, inflammation, and low functional strength, with sleep duration increasing the odds and a healthy lifestyle decreasing the risk. Urine metabolomics revealed differences in enrichment analyses. No significant differences were observed in the Chao1 and InVSimpson indices between the groups. At the genus level, some species were associated with daily sports activity, whereas others were associated with HGS measurements. *Clostridium XIVa* was found only in high- and medium-HGS groups, while *Alistipes*, *Odoribacter*, and *Streptococcus* decreased with activity. Thus, tailored lifestyle interventions may reduce the risk of poor functional strength.

**Supplementary Information:**

The online version contains supplementary material available at 10.1038/s41598-025-02519-5.

## Introduction

Physical activity is defined as any movement of the body via skeletal muscle contractions that results in energy expenditure. In daily life, it can be categorized as occupational, sports, household, or other activities. Skeletal muscle function depends on protein and energy metabolism, as well as various exogenous factors such as diet, alcohol consumption, and smoking^1,2^. Muscular strength is more predictive of adverse events than muscle mass. Handgrip strength (HGS) is a simple and inexpensive non-invasive method for measuring muscular strength and function^3^.

In addition to skeletal muscle mass, the intestine is a central organ for maintaining health and has been the focus of research worldwide^4^. Gut microbiota shifts have been associated with intestinal diseases, systemic inflammation, metabolic and immune diseases, and extraintestinal organ diseases^4,5^. In the research field of gut-organ axes, the hypothesis of the existence of a “gut-muscle axis” is a relatively new term that has been increasingly investigated over the last decade^6,7^. Several studies have provided evidence of a link between the gut microbiota and skeletal muscle mass, strength, and function, with special regard to age-related loss of muscle mass, strength, and function^3^. In a large Chinese population-based observational study, 10% of participants met the sarcopenia diagnostic criteria and had altered gut microbiota composition and function compared to non-sarcopenic participants^8^. This was also observed in a Korean cohort of 1,052 middle-aged individuals. Men with the highest relative muscular masses had significantly higher alpha diversity than those with the lowest^9^, and in the ELDERMET cohort, Claesson et al.^10^ showed that faecal microbiota species richness in older subjects was inversely related to physical performance.

Physical fitness and different activity regimes are related to changes not only in the microbiota but also in metabolomics (for example, SCFA or bile acids)^11–13^. Therefore, the main aim of this study was to examine the role of gut microbiota and its composition to functional strength (muscle strength and physical activity) and to determine potential modifiable predictors for the development of decreased functional strength.

## Methods

### Participants and study design

The Food Chain Plus (FoCus) cohort was randomly recruited between 2011 and 2014 via local registration offices and the Obesity Centre of the University Hospital Schleswig-Holstein (UKSH) in Kiel for population-based research focusing on metabolic inflammation. The study was retrospectively registered under the clinical trial number DRKS00005285 (5th of November 2013) at the German Clinical Trials Register in Cologne. A total of 1,795 participants were recruited, and their medical, anthropometric, sociodemographic, lifestyle, activity, and nutritional data were collected^14^. For secondary analyses, a subcohort of 627 participants (394 women and 233 men, sex assigned at birth) with complete data (phenotype, nutrition, and gut microbiota) was stratified into six functional strength groups depending on their weekly sports activity and handgrip strength (HGS). We defined functional strength as the ability to perform everyday physical activities using strength and physical activity in combination. The combination of these two parameters resulted in six groups for further analysis (see Supplementary Table S1 in the Electronic Supplementary Material).

### Assessment of activity and strength

Weekly activity was assessed using a self-report questionnaire stratified into everyday activities, including walking, housework, gardening, DIY, and sports (see Supplementary Information in the Electronic Supplementary Material). Activity was assumed to be of moderate intensity, as it was not recorded in metabolic equivalents or with an approved activity tracker. Participants reporting ≥ 150 min/week of sports activity were labelled ‘higher activity’ (H), and those reporting less than 150 min/week were labelled ‘lower activity’ (L), following the WHO recommendations^15^. A total of 358 (57%) and 269 (43%) participants were assigned to the H-and L groups, respectively. Subsequently, sports activity is referred to as physical activity (PA).

Isometric handgrip strength (HGS) was measured in kilograms (kg) using a handgrip dynamometer (MAP 80K1; Kern & Sohn GmbH, Balingen, Germany), and the maximum HGS of the dominant hand was used for further analysis. Sex-specific tertiles were calculated and labelled “high” (H) with 206 (33%), “medium” (M) with 202 (32%) and “low” (L) with 219 (35%) participants. Data on sports activity and HGS were combined into six groups (HH, HM, HL, LH, LM, and LL).

### Assessment of dietary intake and healthy lifestyle

#### Adapted dietary inflammatory index (ADII)

Dietary habits were assessed using the European Prospective Investigation into Cancer and Nutrition (EPIC) Potsdam Frequent Food Questionnaire (FFQ), and the average intake of nutritional compounds and food groups was extrapolated from the given information^14^. Participants who reported unrealistic energy intakes (under- and over-reporting) were excluded from the analysis^16^. Nutritional components were energy-adjusted using the residual method described by Willett et al.^17^. The caffeine content of coffee and tea was not included in the dietary composition table and was calculated as described by van Woudenbergh et al.^18^. In the second step, the adapted dietary inflammatory index (ADII) was calculated as described by van Woudenbergh et al.^18^. A higher ADII score indicates a pro-inflammatory diet.

#### Healthy lifestyle score (HLS)

Healthy lifestyle scores were calculated based on the modifiable lifestyle factors. Dichotomized lifestyle factors were used as described by Patel et al.^19^: BMI < 25 kg/m² (1)/ BMI ≥ 25 kg/m² (0), non-current (1)/current (0) smoking, active (1)/non-active (0) (using data from high and low sports activity), low ADII (1)/high ADII (0), and one drink (1)/> 1 drink of alcohol (0) per day. Each participant was scored from 0 (low HLS score) to 5 (high HLS score). This indicates better adherence to a healthy lifestyle with a higher HLS score. The HLS scores calculated in this study ranged from 1 to 5, providing a comprehensive measure of the participants’ lifestyle habits.

### Biomaterial data

Blood samples were collected, and metabolic and inflammatory markers were analyzed as previously described^14^. Stool samples for 16 S rRNA gene sequencing were stored at -80 °C until further processing by the Institute for Clinical Molecular Biology (IKMB) of the UKSH in Kiel. Intestinal microbiota analysis was performed as previously described by Heinsen et al.^20^ and was based on amplicon sequence variants (ASV)^21^. Metabolomic preparation and profiling were performed using serum and spot urine samples obtained from the Division of Food Technology of Kiel University. Samples were prepared and extracted according to the protocols described by Demetrowitsch et al.^22^ and Jensen-Kroll et al.^23^. A targeted panel of nine bile acids in the blood serum was analyzed at the Bremen Medical Laboratory (www.mlhb.de) using high-performance liquid chromatography coupled with mass spectrometry. Missing values were imputed using either LOD (> 50% missing data) or KNN (< 50% missing data). For further information, please see the Supplementary Information regarding the methods used in the Electronic Supplementary Material.

### Statistical analysis

All statistical analyses were performed using R version 4.3.2 and RStudio version 2023.12.1. Data were analyzed in total, stratified by sex (as assigned at birth), and presented as median and interquartile range (IQR,25th + 75th percentile). Categorical variables are expressed as absolute and relative shares (n/N (%)). The Kruskal-Wallis test was used for overall comparisons, and the Wilcoxon test was used with HH as the reference group. Categorical variables were compared using Pearson’s chi-square test and Fisher’s exact test for binary comparisons of categorical variables. Multiple testing corrections (p_adjust_) were calculated using the false discovery rate (FDR). Figures were created using *ggplot2* (version 3.4.4) and *ggstatsplot* (version 0.12.2) in R. Correlation (adjusted and partial) analyses were performed using *corrplot* (version 0.92) and *psych* (version 2.4.1) packages in R. Binomial logistic regression models (univariate and multivariate) were computed to identify potential predictors affecting the probability of being classified as having low functional strength (LL) compared to high functional strength (HH) using the *finalfit* package (version 1.0.7) in R. Previously, significant parameters from microbiota analyses and confounding variables were included as predictors. The results are presented as odds ratios (ORs) with 95% confidence intervals (CIs). Statistical significance was set at *p* < 0.05.

#### Statistical analysis of the gut microbiota

Statistical analysis of the gut microbiota was performed using the R packages *phyloseq* (version 1.46.0), *microbiome* (version 1.24.0), *vegan* (version 2.6.4), and *pscl* (version 1.5.9). ASVs with zero abundance in all samples and samples that lacked microbiota data were excluded. The final pruned dataset contained 12,210 taxa from 578 samples. Alpha (Chao1 and InvSimpson indices) and beta (Bray‒Curtis and Jaccard) diversities were calculated. For alpha diversity, the Kruskal-Wallis test was used for the overall comparison of groups, and the Wilcoxon test was used for comparison with the reference HH and FDR correction for multiple testing. For beta diversity, differences in distances (similarities or dissimilarities) between groups were tested by running a permutational analysis of variance (PERMANOVA) with 1,000 permutations using the *adonis2* function. Additionally, the tests were adjusted for previously determined confounding variables using the *CAPscale* function. Differences were considered statistically significant at *p* < 0.05. The core measurable microbiota (CMM) was determined using the mean and relative abundance of the detected ASVs and examined across different taxonomic levels. The relative ASV abundance in each group was tested against the reference HH.

#### Statistical analysis of metabolomics

Serum and urine metabolomic analyses were performed using *MetaboAnalystR* (version 4.0.0) and *mixOmics* (version 6.26.0). The data were normalized and Pareto-scaled for analysis using *mixomics*. Sparse partial least squares discriminant analysis (sPLS-DA) was used to classify categorically labelled data, and the top 20 metabolites were analyzed in serum and urine samples. The Kruskal-Wallis test with FDR correction was used to test for significant differences between groups (*p* < 0.05).

## Results

### Participant characterization

In total, 627 participants (394 women/233 men) were included in this study (Table 1 and Supplementary Table S2 in the Electronic Supplementary Material). The median age was 52.0 (43.0, 62.5) years, and men were significantly older than women (p_adjust_ < 0.001). Women had a significantly higher BMI than men (p_adjust_ = 0.006). Participants had a median HGS of 28.8 (23.1, 39.0) kg, while women had a lower maximum HGS than men (p_adjust_ < 0.001), and the median sports activity was 165.0 (30.0, 311.25) minutes per week.

**Table 1:** Characterization of the FoCus sub-cohort according to demographic, anthropometric and grouping variables (*n* = 627) ^*^.

Variable	Overall (*n* = 627)	Women (*n* = 394)	Men (*n* = 233)	*p* – Values ^a^	*p*_adjust_ – Values ^b^
Age, y	52.0 (43.0, 62.5)	50.0 (41.0, 60.0)	55.0 (47.0, 65.0)	< 0.001	< 0.001
Age group, n (%)				0.010^†^	0.017^†^
Younger adults, < 65 years	497 (79)	325 (82)	172 (74)		
Older adults, ≥ 65 years	130 (21)	69 (18)	61 (26)		
Height, cm	172.0 (166.0, 180.0)	168.0 (164.0, 172.0)	180.5 (176.0, 185.0)	< 0.001	< 0.001
Weight, kg	95.7 (78.6, 122.1)	93.0 (72.9, 123.9)	97.2 (84.9, 119.9)	0.004	0.008
BMI, kg/m²	31.3 (26.4, 41.4)	33.2 (26.5, 44.1)	29.6 (26.4, 36.5)	0.003	0.006
BMI group, n (%)				< 0.001^†^	< 0.001^†^
Normal weight	119 (19)	77 (20)	42 (18)		
Overweight	154 (25)	75 (19)	79 (34)		
Obesity class I	107 (17)	65 (16)	42 (18)		
Obesity class II	67 (11)	43 (11)	24 (10)		
Obesity class III	180 (29)	134 (34)	46 (20)		
Handgrip strength, kg	28.8 (23.1, 39.0)	24.9 (20.7, 28.8)	43.1 (36.7, 48.1)	< 0.001	< 0.001
Sports, min/week	165.0 (30.0, 311.25)	150.0 (30.0, 300.0)	180.0 (22.5, 352.5)	0.35	0.43
Activity group, n (%)				0.99	0.99
HH	126 (20)	77 (20)	49 (21)		
HM	105 (17)	64 (16)	41 (18)		
HL	127 (20)	81 (21)	46 (20)		
LH	80 (13)	52 (13)	28 (12)		
LM	97 (15)	62 (16)	35 (15)		
LL	92 (15)	58 (15)	34 (15)		

^*^Values are shown as median (interquartile range) or n (%).

^a, b^p-values were derived from Wilcoxon rank sum test unless otherwise indicated and were adjusted for multiple testing using False discovery rate correction.

^†^From Pearson’s Chi-squared test.

Adjust = adjusted; BMI = body mass index; HH = high sports activity and high handgrip strength; HM = high sports activity and medium handgrip strength; HL = high sports activity and low handgrip strength; LH = low sports activity and high handgrip strength; LM = low sports activity and medium handgrip strength; LL = low sports activity and low handgrip strength.

### Clinical characteristics and functional strength

#### Phenotypic, metabolic and inflammatory marker

**Fig. 1:**
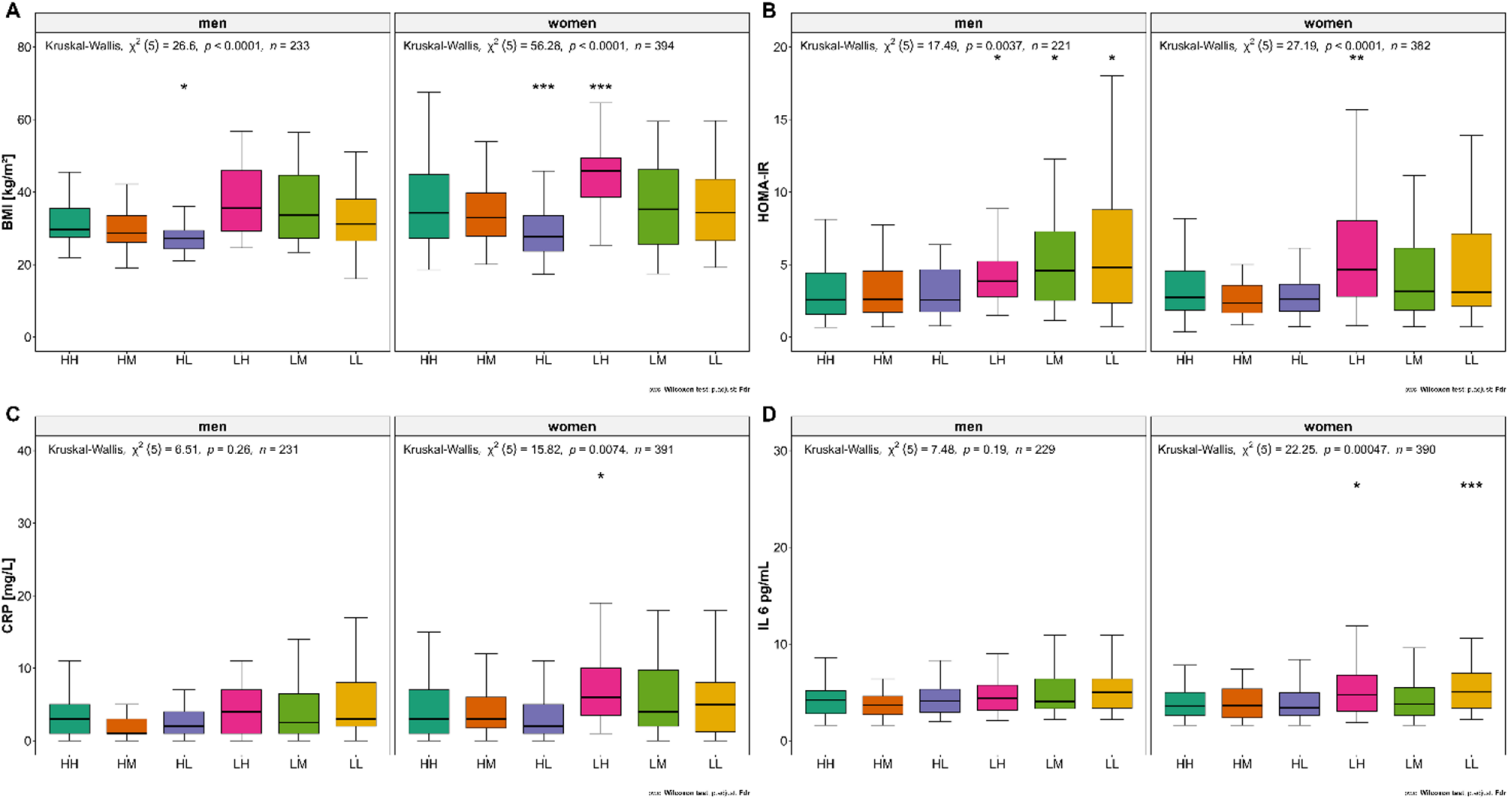
Metabolic and inflammatory markers across the functional strength groups (*n* = 627). Differences between women and men for functional strength groups in (**A**) Body Mass Index (BMI), (**B**) Homeostatic Model Assessment of Insulin Resistance (HOMA-IR), (**C**) C-reactive protein (CRP), and (**D**) Interleukin-6 (IL-6). P-values were calculated using the Kruskal-Wallis rank sum test for overall comparison with FDR correction for multiple testing and Wilcoxon test with FDR correction for multiple testing for comparison with the reference group HH (significance cut-off points: *p ≤ 0.05, **p ≤ 0.01, *** *p* ≤ 0.001, ****p ≤ 0.0001). HH, high sports activity and high handgrip strength; HM, high sports activity and medium handgrip strength; HL, high sports activity and low handgrip strength; LH, low sports activity and high handgrip strength; LM, low sports activity and medium handgrip strength; LL, low sports activity and low handgrip strength.

BMI differed significantly between women and men in the overall group comparison (Fig. 1A). Participants of both sexes with lower functional strength tended to have a higher BMI. HOMA-IR differed significantly in the overall comparison between sexes (Fig. 1B), and higher HOMA-IR levels were associated with the functional strength groups. CRP levels differed significantly between the groups (p_adjust_ = 0.0074) in women but not in men (Fig. 1C). CRP levels were significantly higher in the LH group than in the HH group (*p* < 0.05) (Fig. 1C). Interleukin- 6 (IL 6) levels differed significantly between the groups (p_adjust_ = 0.00047) in women. When compared to HH, the median IL-6 levels were significantly higher in LH (*p* < 0.05) and LL (*p* < 0.05) (Fig. 1D). Of the nine different bile acids, only deoxycholic acid (DC) was nominally significantly different across the groups (*p* < 0.05), with the lowest levels of deoxycholic acid in the LH and HH groups.

#### Health status, healthy lifestyle and functional strength

**Fig. 2:**
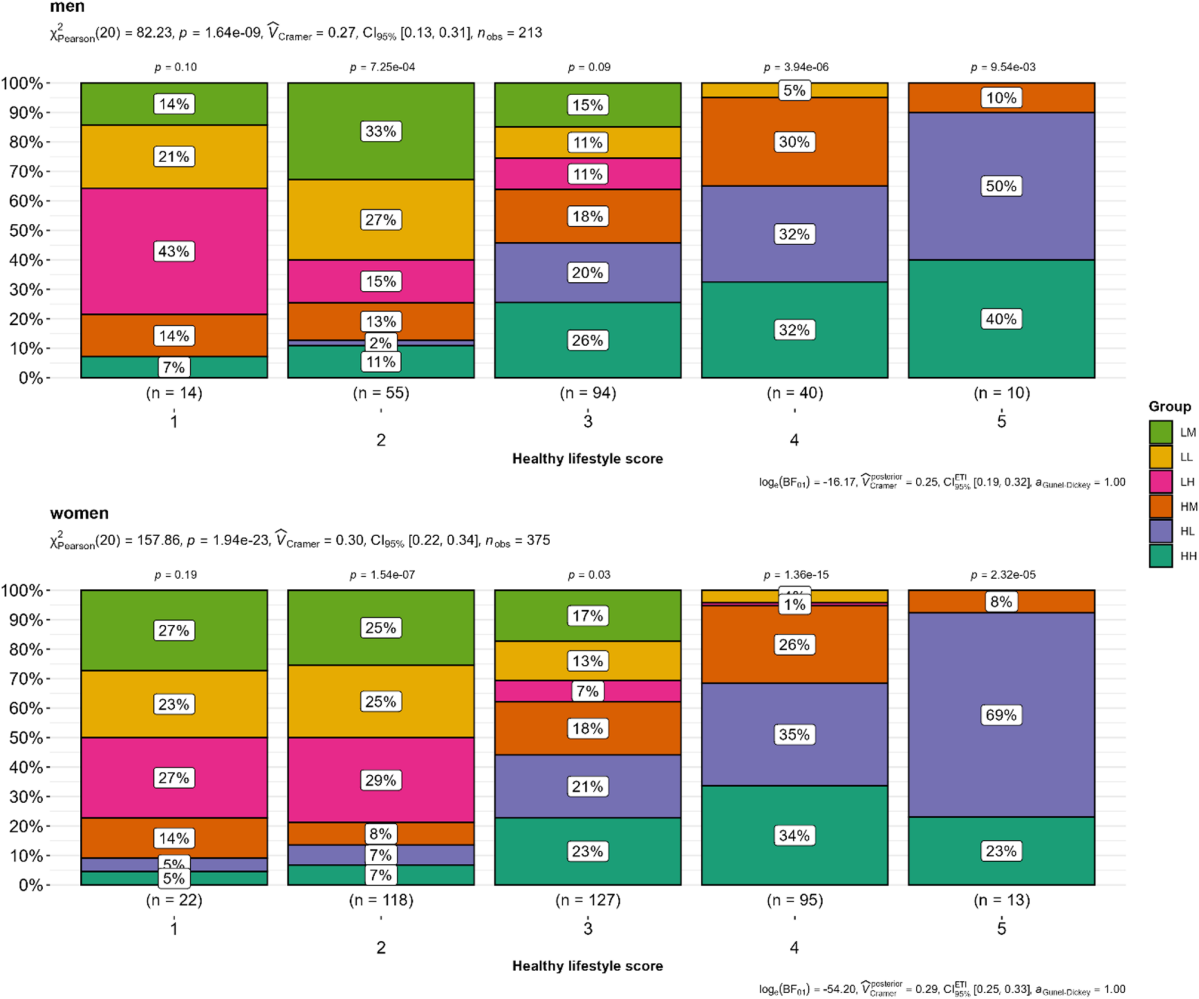
Comparison of healthy lifestyle scores (HLS) between functional strength groups in women and men (*n* = 588). A higher HLS score indicates better adherence to a healthy lifestyle. The HLS scores calculated in this study ranged from 1 (low adherence) to 5 (high adherence). P-values were calculated using Pearson’s chi-squared test for overall score and group comparison, and within a single score and group comparison. HH, high sports activity and high handgrip strength; HM, high sports activity and medium handgrip strength; HL, high sports activity and low handgrip strength; LH, low sports activity and high handgrip strength; LM, low sports activity and medium handgrip strength; LL, low sports activity and low handgrip strength.

A total of 470 (75%) participants reported regular medication use, and group comparisons showed a significant difference (p_adjust_ = 0.005). The prevalence of hypertension was significantly different between the groups (p_adjust_ < 0.001). Overall, 50% of the participants had a history of hypertension and 18% (111/627) had a history of diabetes (Supplementary Table S3 in the Electronic Supplementary Material). 20% of the participants (124/627) were current smokers. Daytime sleep duration was significantly different among the groups (p_adjust_ < 0.001). The total activity was not significantly different between the groups (p_adjust_ = 0.42; see Supplementary Table S4 in the Electronic Supplementary Material). The healthy lifestyle scores of the different groups were analyzed and are shown in Fig. 2. The overall HLS was compared between women and men using Pearson’s chi-square test. HLS was compared between the groups using the same test. Overall, the prevalence of HLS scores differed significantly between the functional strength groups in both sexes (*p* < 0.05), and participants with higher activity levels showed higher adherence to a higher HLS (scores 4 and 5), independent of their HGS (Fig. 2).

#### Adapted dietary inflammatory index, tryptophan intake and functional strength

Supplementary Table S4 (in the Electronic Supplementary Material) shows that the ADII was significantly different between the groups in women (*p* = 0.0038) but not in men. In women, there was a tendency to have a more proinflammatory ADII in the lower functional strength groups. The relative amount of tryptophan did not differ between the groups for either sex.

#### Gut microbiota and functional strength

**Fig. 3:**
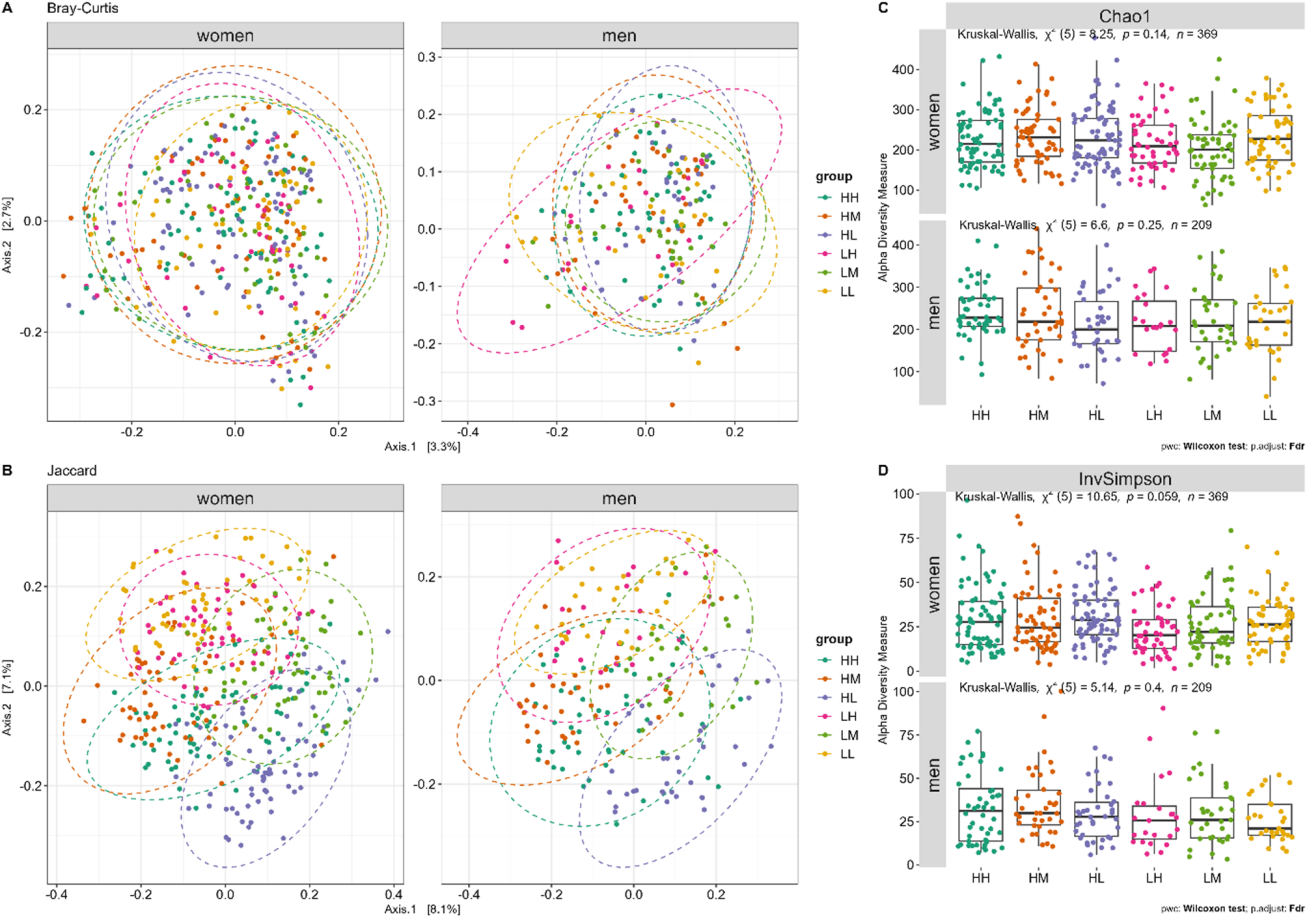
Differences in beta (ß) and alpha (α) diversity indices for functional strength groups (*n* = 578) in women and men. Differences are shown for (**A**) ß-diversity using the Bray-Curtis dissimilarity metric, (**B**) ß-diversity using the Jaccard similarity index, (**C**) α-diversity measured by the Chao1 richness estimator, and (**D**) α-diversity measured by the Inverse Simpson (InvSimpson) index. ß-diversity plots (**A** and **B**) illustrate community compositional differences between groups, whereas α-diversity indices (**C** and **D**) represent species richness and evenness within each group. In A and B, each point represents an individual sample, with colors denoting different functional strength groups. Ellipses indicate the 95% confidence intervals of each group. P-values were calculated using PERMANOVA (1,000 permutations and significance level set at *p* < 0.05) for ß-diversity and Kruskal-Wallis rank sum test for overall comparison with FDR correction for multiple testing for alpha diversity and Wilcoxon test with FDR correction for multiple testing for comparison with the reference group HH (significance cut-off points by *p ≤ 0.05, **p ≤ 0.01, ***p ≤ 0.001, ****p ≤ 0.0001). HH, high sports activity and high handgrip strength; HM, high sports activity and medium handgrip strength; HL, high sports activity and low handgrip strength; LH, low sports activity and high handgrip strength; LM, low sports activity and medium handgrip strength; LL, low sports activity and low handgrip strength.

Distance plots by Bray‒Curtis and Jaccard are presented in Fig. 3A and B, with significant group differences for the unadjusted model (Bray‒Curtis, *p* = 0.039 (men); Jaccard, *p* = 0.035 (men)). After adjusting for confounders (age, HOMA-IR, CRP, IL-6, regular medication, night and day sleep, HLS, and sex), PERMANOVA revealed significant differences in beta diversity between groups in men for Bray-Curtis (*p* = 0.0489) and Jaccard’s similarity coefficient (*p* = 0.0379). The Chao1 and InvSimpson indices were not significantly different between the groups (Fig. 3C and D) for both sexes.

Comparisons of CMM revealed potential sports activity- or HGS-related differences in the relative abundance at the genus level (see Supplementary Fig. S1 in the Electronic Supplementary Material). *Akkermansia and Odoribacter* were reduced in the low-activity group, independent of the HGS tertiles, and differed significantly from those in the other groups (*p* < 0.0001). The abundance of *Streptococcus* was reduced in the LH and LL groups. In contrast, taxa of the genus *Fusicatenibacter* were abundant only in the LH and LM groups but not in the higher sports activity groups and the LL group. Taxa of the *Parasutterella* genus were abundant only in the HH and LH groups with high HGS. *Blautia* abundance tended to be lower in those with lower HGS and decreased independently of activity level (*p* < 0.0001). Subsequently, analyses were performed for both the sports activity and HGS groups (Supplementary Figs. S2 and S3, respectively (Electronic Supplementary Material). *Clostridium XIVa*, *Parasutterella*, and *Dialister* in men were only associated with HGS and showed reduced abundance with reduced HGS (see Supplementary Fig. S2 in the Electronic Supplementary Material). *Alistipes* abundance was reduced only in women with lower activity levels, whereas *Odoribacter* and *Streptococcus* abundance was reduced in both sexes with lower activity levels. In contrast, the abundance of *Blautia* increased at lower activity levels in both sexes (Supplementary Fig. S3 in the Electronic Supplementary Material).

#### Metabolomics and functional strength

Two sPLS-DA models were fitted with two components in the serum and urine samples from women and men. Neither component 1 nor component 2 clearly distinguished between the groups for either sex. This was true for both the serum and urine metabolites. Enrichment analysis using HMDBs from significantly (p_adjust_ < 0.1) different serum and urine metabolites of both components was matched to terms in the SMPDB database in *MetaboAnalyst*. The results are presented in Supplementary Table S5 (Electronic Supplementary Material). Only caffeine metabolism was significant in the enrichment and pathway analyses of urine metabolomics in women, even after FDR correction (Supplementary Fig. S4 in the Electronic Supplementary Material).

####  Predictive variables for low functional strength

**Fig. 4:**
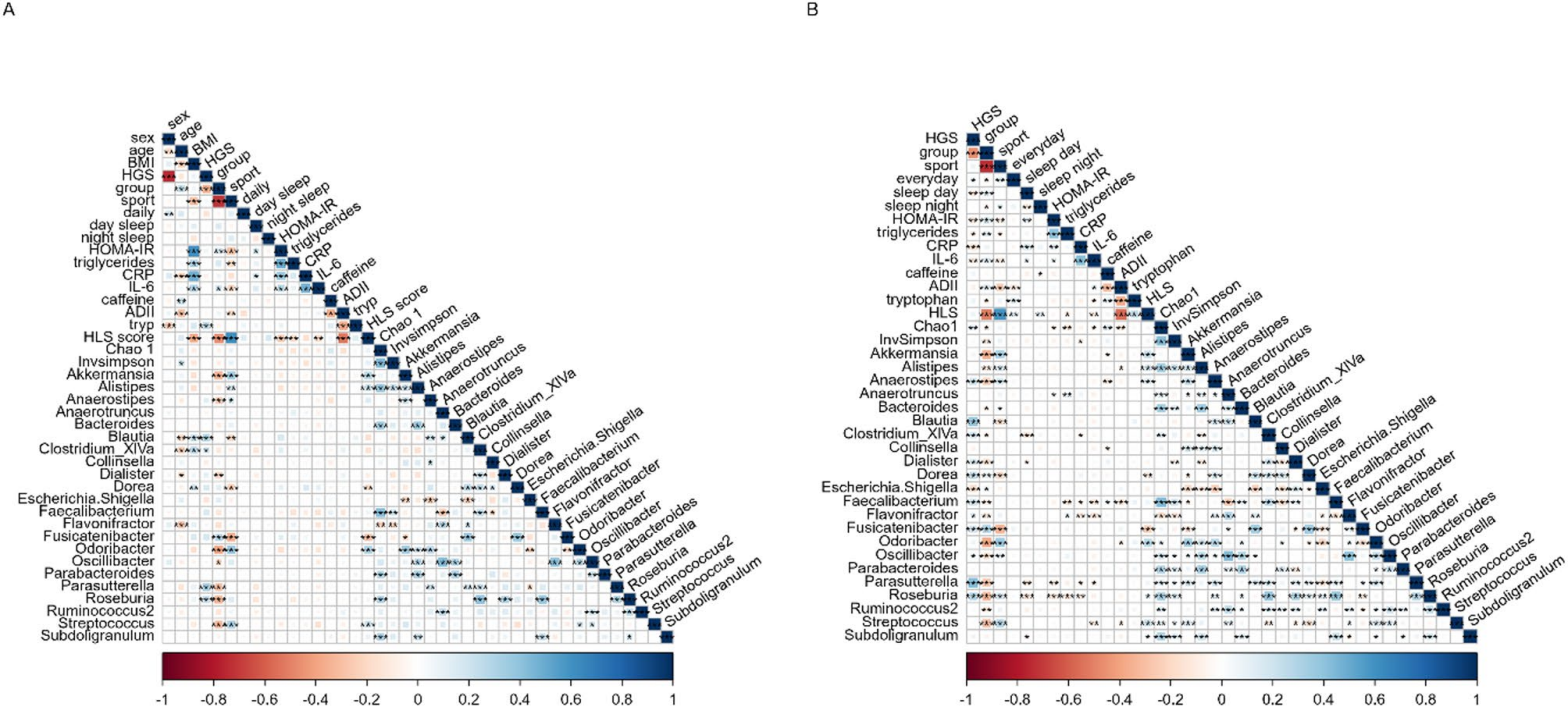
Correlation plots showing (**A**) the correlations adjusted for multiple testing, and (**B**) the correlations adjusted for age, sex, and BMI between handgrip strength (HGS), sports activity, strength fitness groups, inflammatory and lifestyle parameters, and relative microbial abundance. Blue and red indicate positive and negative correlations, respectively. The color density and square size reflect the scale of the correlation significance cut-off points by *p ≤ 0.05, **p ≤ 0.01, ***p ≤ 0.001, and ****p ≤ 0.0001. CRP, C-reactive protein; IL-6, Interleukin 6; HLS, healthy lifestyle score; ADII, adapted dietary inflammatory index.

Correlation analysis was performed using the data from the previous analysis, and Fig. 4A and B show the results of the adjusted and partial correlation analyses, respectively. Correlation analysis showed that after correction for multiple testing, sports activity in minutes per week was inversely related to metabolic biomarkers and positively related to healthy lifestyle and to tryptophan intake. Sports activity was also related to *Akkermansia*,* Alistipes*,* Bacteroides*,* Odoribacter*,* Oscillibacter*, and *Streptococcus.* Absolute HGS in was more strongly related to sex, age, tryptophan intake, and the taxa *Clostridium XIVa*, *Dialister*,* Escherichia.Shigella*,* Faecalibacterium* and *Parasutterella*. Caffeine intake was inversely correlated with the ADII, *Anaerostipes*, and *Flavonifractor* and positively correlated with the Chao1 and InvSimpson indices. This was also true after adjusting for sex, age, and BMI.

Supplementary Table S6 (in the Electronic Supplementary Material) presents the odds ratios (OR) with 95% confidence intervals (CI)) of the potential predictors of low functional strength using a multivariate binomial logistic regression model with functional strength groups. Significant predictors associated with an increase in the probability of low functional strength (LL) were age (OR (95% CI) 1.12 (1.08–1.17, *p* < 0.001)) and daytime sleep (OR (95% CI), 2.72 (1.51–5.42, *p* = 0.002)), whereas high HLS adherence reduced the risk of low functional strength (OR (95% CI) 0.18 (0.09–0.33, *p* < 0.001)). Supplementary Table S7 (in the Electronic Supplementary Material) presents the odds ratios of potential predictors, including microbiota, for the likelihood of having a low functional strength level and shows that the inclusion of the Chao1 and InvSimpson indices did not significantly change the previously mentioned confounders.

## Discussion

This study identified several key determinants of functional strength through a cross-sectional analysis. Age, inflammation markers, and daytime sleep duration were associated with increased odds of low functional strength, whereas higher adherence to a healthy lifestyle score reduced these odds. The gut microbiota β-diversity differed significantly between groups in men, and certain gut microbiota taxa were associated with sports activity level or handgrip strength. Caffeine metabolism pathways were enriched in the urine metabolomics of women. Multivariate analysis confirmed that age, healthy lifestyle score, and daytime sleep were significant predictors of strength fitness. While these findings suggest potential targets for tailored lifestyle interventions to improve functional strength, longitudinal data are needed to confirm these associations. These findings highlight the importance of lifestyle factors in determining functional strength and suggest potential avenues for intervention, particularly when these results are compared with those of previous surveys. A German survey revealed that only 29.3% of men and 21.6% of women were active in sports^24^. In contrast, nearly 60% of the participants were classified as active. In a large-scale study, HGS was found to be positively associated with BMI in men^25^. As expected, participants with higher sports activity levels had a lower median BMI than those with lower sports activity levels.

A healthy lifestyle score was associated with a reduced risk of lower functional strength. This was also shown by Sternfeld et al.^26^ in a study of midlife women, in which the authors reported that a higher HLS was associated with better physical activity and more significantly associated with physical performance than with strength alone. This was also observed in the present study (data not shown). In contrast, a study on cardiorespiratory fitness in adults aged 18–70 years showed that HLS was related to muscle strength, as measured by HGS^27^.

The alpha and beta diversity results were comparable to earlier findings from a large population-based study of participants aged ≥ 50 years, in which alpha diversity did not differ between sarcopenic and non-sarcopenic participants; however, beta diversity differed between these groups^8^. In a smaller study, patients with (pre) sarcopenia showed reduced alpha and beta diversities compared with controls^28^. A cross-sectional study of 207 community-dwelling older adults (> 65 years) showed higher gut microbiota diversity in high-fitness phenotypes than in low-fitness phenotypes^29^.

The relative abundances of several taxa were associated with either sports activity or HGS. *Akkermansia*,* Alistipes*,* Bacteroides*,* Odoribacter*,* Osicillibacter*,* and Streptococcus* were activity-related. Most of these are SCFA producers. For example, *Akkermansia muciniphila* has been observed to be more abundant in active women than in inactive women^30^ or in athletes with a lower BMI^31^. These findings support the activity-related differences observed in the abundance of this species. *Erysipelotrichaceae* abundance has previously been described to be higher with a higher fitness level than with a lower fitness level, accompanied by higher fecal butyrate levels, and *Clostridiales*, *Roseburia*, and *Lachnospiraceae* have been elevated with high fitness levels^32^. *Clostridium XIVa*,* Dialister*,* Escherichia.Shigella*,* Faecalibacterium*, and *Parasutterella* are closely related to HGS. *Anaerostipes hadrus* and *Faecalibacterium prausnitzii* are well-known butyrate producers, and both species, among other butyrate producers, have been combined into a total relative abundance of butyrate-producing bacteria that has been positively associated with muscle health (mass, strength, and function)^33^. Relative abundance comparisons between groups showed differences in the genus *Parasutterella*, which was only abundant in the groups with high HGS. In a previous study by our group, *Parasutterella* was positively associated with obesity, diabetes, carbohydrate intake, and activation of the fatty acid biosynthesis pathway, suggesting that it promotes weight gain^34^. *Dialister invisus* has been shown to shares the same fuel (L-cysteine) as *Parasutterella*^34^.

Enrichment analyses showed that, in women, only caffeine metabolism was significantly affected, even after false discovery rate (FDR) correction. Caffeine supplementation is a popular strategy for improving physical performance in sports. Giráldez-Costas et al.^35^ showed that pre-exercise caffeine intake induced more muscle performance adaptations than strength improvements, using bench press and force velocity tests. In the correlation and logistic regression analyses, caffeine intake was not related to either HGS or activity. Targeted metabolomic analyses revealed that deoxycholic acid (DC) levels were lower in the high-HGS group, which contrasts with recent findings. However, recent publications have also shown that bile acids are regulated differently by resistance or endurance training, depending on the time^11–13^.

A multivariate binomial logistic regression model revealed significant effects of age, HLS, and daytime sleep on functional strength. However, the microbiota data showed no significant effect. Age was associated with a 2.12-fold increase in the risk of developing sarcopenia^36^. Similarly, an independent study found a 20% higher risk of developing sarcopenia (examined using HGS) over eight years^37^. Sarcopenia is associated with inflammation and impairment of mitochondrial and metabolic functions^38^. In the present study, C-reactive protein levels had a nearly significant effect on increasing the probability of low functional strength. Daytime sleep was significantly associated with a 2.72-fold probability increase in low functional strength. Physical inactivity has been well investigated as a risk factor for sarcopenia and may be linked to longer daytime sleep duration. Findings from a British longitudinal study of older adults emphasized the positive effect of physical activity on sarcopenia risk^37^.

## Conclusion

The participants showed several group differences that might be related to their sports activity level or their maximum handgrip strength. Participants in the low HGS group were older and had the lowest BMIs within the same sports activity group. Metabolic and inflammatory markers, prevalence of regular medication, obesity, hypertension, diabetes, smoking, daytime sleep, and HLS were more likely to be associated with sports activity levels.

Regarding microbiota diversity, inter-individual differences (beta diversity) have been suggested to play a greater role in reduced functional strength than reduced species richness (alpha diversity). Taxa abundance in the CMM also showed differences that might be related to sports activity or HGS. As expected, age was a non-modifiable predictor of low functional strength. With a small but not significant effect size, C-reactive protein level also proved to be a potential modifiable predictor. Daytime sleep showed the highest potential for predicting low functional strength compared to high functional strength. Longitudinal data are required to confirm the potential of these predictors. Unfortunately, the microbiome data did not reveal viable outcomes in the logistic regression model, and the Chao1 and InvSimpson indices showed inconclusive results. However, beta diversity and some taxa tended to play a role in the low functional strength. For future research, the integration of multiple datasets (e.g., phenotype, biomarker, and omics data) in supervised analysis is necessary to reveal host-omics interactions in subjects at risk of low functional strength, with the option of targeted lifestyle interventions that also work at the omics level to reduce the risk of frailty and sarcopenia.

### Strengths and limitations

Data on the participants’ dietary intake were based on a self-reported semiquantitative food frequency questionnaire (FFQ). Participants needed to have a very good memory and be able to correctly estimate portion sizes, which could lead to over- or under-reporting of their dietary habits. These participants were excluded from the ADII analysis. A limitation of this study is that we did not directly measure the intensity of sports activities or use an approved activity tracker to quantify the metabolic equivalents (METs). Instead, we relied on self-reported data on weekly sports activity duration and assumed a moderate intensity level for all the participants. There was also a risk of over- and under-reporting of physical activity, including weekly exercise, which was used as a grouping variable. Future studies could benefit from incorporating objective measures of physical activity intensity, such as wearable devices and validated questionnaires. This would provide more accurate data on METs and allow for a more nuanced analysis of the relationship between sports activities and health outcomes. As all analyses were cross-sectional, it was only possible to detect differences between groups and associations with the analyzed parameters, but not causality.

However, this study had several strengths. Participants in the FoCus cohort were assessed for a wide range of parameters, including anthropometric data, health and socioeconomic status, lifestyle and activities, dietary habits, microbiome, metabolome, and genetic data.

## Electronic supplementary material

Below is the link to the electronic supplementary material.

Supplementary Material 1

Supplementary Material 2

Supplementary Material 3

## Data Availability

Data and biomaterials are stored at the biobank P2N and can be requested there (https://portal.popgen.de).
