# Supplementary material for: Healthy lifestyle, daytime sleepiness, and gut microbiome composition are determinants of functional strength in humans: a cross-sectional study
Source: Sci Rep. 2025 May 19;15:17378. doi: 10.1038/s41598-025-02519-5 (PMC12089321; doi:10.1038/s41598-025-02519-5)
Supplement: Supplementary file 1 — Supplementary Material 1 [file 41598_2025_2519_MOESM1_ESM.docx]

**Healthy lifestyle, daytime sleepiness, and gut microbiome composition are determinants of functional strength in humans: a cross-sectional study**

Friederike Norkeweit^1^, Kristina Schlicht^1^, Nathalie Rohmann^1^, Katharina Hartmann^1^, Kathrin Türk^1^, Ute Settgast^2^, Dominik M. Schulte^1,2^, Felix Gilbert^3^, Tobias Demetrowitsch^4^, Fynn Brix^4^, Corinna Bang^3^, Andre Franke^3^, Karin Schwarz^4^, Matthias Laudes^1,2^ and Corinna Geisler^1,^*

^1^Institute of Diabetes and Clinical Metabolic Research, University Medical Center Schleswig-Holstein and Kiel University, Kiel 24105, Germany

^2^Division of Endocrinology, Diabetes and Clinical Nutrition, Department of Internal Medicine I, University Medical Center Schleswig-Holstein, Campus Kiel, Kiel 24105, Germany

^3^Institute of Clinical Molecular Biology (IKMB), Kiel University, Kiel 24118, Germany

^4^Division of Food Technology, Institute of Human Nutrition and Food Science, Kiel University, Kiel 24105, Germany

*Address correspondence to: Corinna Geisler, PhD, PD; Institute of Diabetes and Clinical Metabolic Research, University Medical Center, Schleswig-Holstein and Kiel University, Kiel 24105, Germany. E-mail: corinna.geisler@uksh.de; Telephone: +4943150022446

**Supplemental information for Materials and Methods section**

**1. Assessment of Activity**

For health-driven physical fitness, weekly cycling and sports activities were summed up for total sports activity per week. Some activities (walking, cycling, sports, and gardening) were considered separately for summer (April-September) and winter (October-March). The means for both seasons were used.

**2. Assessment of Handgrip Strength**

Participants were asked to sit upright and hold their upper arm and forearm at a right angle. To maintain this position, the participants were required to squeeze the dynamometer as strongly as they could. Grip strength was measured thrice for each hand on a rotating basis ^1^. The participants were asked whether they were left-, right-, or ambidextrous.

**3. Biomaterial Collection**

Fasting blood samples were obtained by venipuncture after overnight fasting for the biochemical analysis of metabolic and inflammatory markers. Midstream urine samples for metabolomics were collected on the day of the visit, and stool samples for microbiome analysis were collected by the participants prior to visit ^2^.

**3.1 Blood sample analyses**

C-reactive protein (CRP), interleukin-6 (IL-6), fasting glucose, fasting insulin, and triglyceride levels were analyzed in the central laboratory of the UKSH in Kiel on the day of collection. HOMA-IR was calculated (Homeostasis Model Assessment Insulin Resistance = fasting glucose (mg/dL) × fasting insulin (μU/mL) / 405) as an index of insulin sensitivity. Blood samples for central laboratory analysis were stored at 4 °C until transport.

All other blood samples and biomaterials were collected and processed according to the internal laboratory standardised operating procedures. The blood samples were centrifuged, separated, and aliquoted. Whole blood samples were frozen for subsequent genotype analysis. Aliquots of the samples were stored at -80 °C.

**3.2 Microbiota analyses**

DNA extraction from stool samples

DNA was extracted from the stool samples using the QIAamp DNA Stool Mini Kit and QIAcube system (both from Qiagen). After thawing, approximately 200 mg of sample material was transferred to bead-beating tubes (Garnet, 0.7 mm) filled with 1.1 ml of ASL lysis buffer. The solution in the tubes was homogenized using SpeedMill PLUS (Analytik Jena) for 45 s at 50 Hz. The samples were then heated to 95 °C for 5 min. All further steps were performed according to the manufacturer's protocol.

Amplicon sequencing of bacterial 16S rRNA and quality control

During preparation for sequencing, the variable V1-V2 region of the 16S rRNA gene was amplified by polymerase chain reaction (PCR), using a pair of primers 27F / 338R with an individual combination of two barcodes according to the dual barcoding approach described by Caporaso et al. ^3^. The SequalPrep Normalization Plate Kit (Thermo Fisher Scientific) was used to normalize the DNA concentration of the PCR products according to the manufacturer’s instructions. This was followed by sequencing of the prepared DNA solution using an Illumina MiSeq device. For this purpose, the individual samples were mixed equimolarly (“pooled”). In the absence of errors, the generated sequences were assigned to the corresponding samples. Complementary sequences were read in the forward and reverse directions and combined using the FLASH program. The Illumina company also provides a Q score for the Miseq device, which is used for quality control. Sequences with a Q-score below 30 in more than 5% of the nucleotides were sorted out using additional software (UCHIME). After quality control, 1,541 samples remained for the statistical analyses.

Taxonomy was assigned using the RDP classifier ^4^, whereby those with a low confidence below 0.8 at the genus level were summarized as “unclassified” (NA) at the family level ^5^. Seven taxonomic levels (domain, phylum, class, order, family, genus, and species) were determined for each ASV, and absolute abundance was calculated by randomly selecting 1,000 sequences from each sample and creating a "taxon-by-sample abundance table" ^5^. The core measurable microbiome (CMM) was determined, and only taxonomic groups with a mean abundance of more than 0.5% of all bacteria per group (detection) and ASVs abundant in at least 40 % of the samples per group (prevalence) were included in the CMM.

**3.3 Metabolomics analyses**

Untargeted metabolomics

Blood samples were extracted according to the protocol described by Jensen-Kroll et al. ^6^. Therefore, 500 µL of ultra-pure methanol (Carl Roth, Germany) and 4 ml of methyl-tert-butylether (LC-grade, Carl Roth, Germany) were added to 100 µL plasma samples. The samples were incubated for 30 min at 25 rpm in an end-over-end shaker. For phase separation, 500 µL of ultrapure water (Carl Roth, Germany) was added and incubated for 10 min without shaking. Subsequently, the samples were centrifuged at 4000 × g and 4 °C for 10 min, and the upper lipophilic phase was collected and stored in another tube. The remaining hydrophilic phase was used as a template to repeat the aforementioned protocol.

After extraction, the hydrophilic and lipophilic phases were dried at 0.1 bar and 45 °C (SpeedVac; Thermo Scientific, Germany). Hydrophilic samples were resuspended in 500 µL of ultrapure water and methanol (50/50, v/v, ultrapure grade, Carl Roth, Germany). Lipophilic samples were prepared in 500 µL of isopropanol and chloroform (3:1, v/v, both LC grade, Carl Roth, Germany).

Finally, both sample phases were diluted 1:1000 with the respective eluents to a final concentration of 1:500,000 and stored at -80 °C until the day of measurement.

Measurements were conducted using an extremely high-resolution Fourier transform ion cyclotron resonance mass spectrometer (7T, SolariXR, 2-omega-cell, Bruker, Bremen, Germany) in direct-injection mode. Therefore, the mass spectrometer was linked to a high-performance liquid chromatography pump and an autosampler (HPLC, 1260 Infinity System, Agilent, Waldbronn, Germany) for automated sample injection without sample chromatography. Data were acquired with an electrospray ionization source in both modes (positive and negative ionization mode) and with a mass range of 65‑1500 Da (for more details, see Seoudy et al. 2023 ^7^). Data were pre-processed using MetaboScape 2021b software (Bruker, Bremen, Germany), including mass calibration based on a customized local matrix database with a value < 0.5 ppm. Metabolite identification was conducted using MetaboScape 2021b with a tolerated mass error of < 1 ppm and an isotopic fine structure error of < 300 mSigma. For annotation, a local database and the Human Metabolome Database v.5.0 ^8^ were used for annotation. For the non-targeted approach, unknown metabolites after database annotation were assigned a calculated sum formula based on accurately measured masses, isotopic fine structure, and the seven golden rules ^9^, whereas compound names were matched to the Human Metabolome Database v.5.0 ^8^. Signal correction, peak filtering, and imputation of missing values were conducted using the R package “statTarget” v.1.24.0 ^10^. Signal correction was performed using a QC-based random forest. Peak filtering was performed by including only compounds that were detected in at least 80 % of the samples. Missing values were imputed using the k-nearest neighbor method. Subsequently, the datasets were merged into a final dataset.

Targeted metabolomics

A comprehensive bile acid panel in blood samples was analyzed by an accredited medical laboratory (Medizinisches Labor Bremen, 28357 Bremen, Germany) using liquid chromatography with tandem mass spectrometry (LC-MS) for the following bile acids: chenodesoxycholic acid, cholic acid, deoxycholic acid, glycocholic acid, glycochenodesoxycholic acid, glycodesoxycholic acid, taurochenodesoxycholic acid, taurocholic acid, tauroursodeoxycholic acid, and ursodeoxycholic acid.

**References**

1. Reijnierse EM*, et al.* Assessment of maximal handgrip strength: how many attempts are needed? *Journal of Cachexia, Sarcopenia and Muscle* **8**, 466-474 (2017).

2. Geisler C*, et al.* Cohort profile: the Food Chain Plus (FoCus) cohort. *European Journal of Epidemiology* **37**, 1087–1105 (2022).

3. Caporaso JG*, et al.* Ultra-high-throughput microbial community analysis on the Illumina HiSeq and MiSeq platforms. *ISME J* **6**, 1621-1624 (2012).

4. Wang Q, Garrity GM, Tiedje JM, Cole JR. Naïve Bayesian Classifier for Rapid Assignment of rRNA Sequences into the New Bacterial Taxonomy. *Applied and Environmental Microbiology* **73**, 5261-5267 (2007).

5. Heinsen F-A*, et al.* Beneficial Effects of a Dietary Weight Loss Intervention on Human Gut Microbiome Diversity and Metabolism Are Not Sustained during Weight Maintenance. *Obesity Facts* **9**, 379-391 (2016).

6. Jensen-Kroll J, Demetrowitsch T, Clawin-Radecker I, Klempt M, Waschina S, Schwarz K. Microbiota independent effects of oligosaccharides on Caco-2 cells -A semi-targeted metabolomics approach using DI-FT-ICR-MS coupled with pathway enrichment analysis. *Front Mol Biosci* **9**, 968643 (2022).

7. Seoudy AK*, et al.* A PROSPECTIVE ANALYSIS OF THE METYRAPONE SHORT TEST USING TARGETED AND UNTARGETED METABOLOMICS. *Neuroendocrinology*, (2023).

8. Wishart DS*, et al.* HMDB 5.0: the Human Metabolome Database for 2022. *Nucleic Acids Research* **50**, D622-D631 (2022).

9. Kind T, Fiehn O. Seven Golden Rules for heuristic filtering of molecular formulas obtained by accurate mass spectrometry. *BMC Bioinformatics* **8**, (2007).

10. Luan H, Ji F, Chen Y, Cai Z. statTarget: A streamlined tool for signal drift correction and interpretations of quantitative mass spectrometry-based omics data. *Analytica Chimica Acta* **1036**, 66-72 (2018).
