# Supplementary material for: Healthy lifestyle, daytime sleepiness, and gut microbiome composition are determinants of functional strength in humans: a cross-sectional study
Source: Sci Rep. 2025 May 19;15:17378. doi: 10.1038/s41598-025-02519-5 (PMC12089321; doi:10.1038/s41598-025-02519-5)
Supplement: Supplementary file 2 — Supplementary Material 2 [file 41598_2025_2519_MOESM2_ESM.docx]

**Healthy lifestyle, daytime sleepiness, and gut microbiome composition are determinants of functional strength in humans: a cross-sectional study**

Friederike Norkeweit^1^, Kristina Schlicht^1^, Nathalie Rohmann^1^, Katharina Hartmann^1^, Kathrin Türk^1^, Ute Settgast^2^, Dominik M. Schulte^1,2^, Felix Gilbert^3^, Tobias Demetrowitsch^4^, Fynn Brix^4^, Corinna Bang^3^, Andre Franke^3^, Karin Schwarz^4^, Matthias Laudes^1,2^ and Corinna Geisler^1,^*

^1^Institute of Diabetes and Clinical Metabolic Research, University Medical Center Schleswig-Holstein and Kiel University, Kiel 24105, Germany

^2^Division of Endocrinology, Diabetes and Clinical Nutrition, Department of Internal Medicine I, University Medical Center Schleswig-Holstein, Campus Kiel, Kiel 24105, Germany

^3^Institute of Clinical Molecular Biology (IKMB), Kiel University, Kiel 24118, Germany

^4^Division of Food Technology, Institute of Human Nutrition and Food Science, Kiel University, Kiel 24105, Germany

*Address correspondence to: Corinna Geisler, PhD, PD; Institute of Diabetes and Clinical Metabolic Research, University Medical Center, Schleswig-Holstein and Kiel University, Kiel 24105, Germany. E-mail: corinna.geisler@uksh.de; Telephone: +4943150022446

**Table S1 Group distribution in sports activity and handgrip strength groups of FoCus cohort participants (n = 627)**

| Group | Sports activity group | HGS group | n (%) |
| --- | --- | --- | --- |
| HH  HM  HL | Higher sports activity (H)  Higher sports activity (H)  Higher sports activity (H) | High (H)  Medium (M)  Low (L) | 126 (20)  105 (17)  127 (20) |
| LH  LM  LL | Lower sports activity (L)  Lower sports activity (L)  Lower sports activity (L) | High (H)  Medium (M)  Low (L) | 80 (13)  97 (15)  92 (15) |
| *Note:* HGS = handgrip strength | | | |

**Table S2 Group comparisons regarding demographic and anthropometric parameter and physical and medical measurements (n = 627)***

| Variable | Overall  n = 627 | HH  n = 126 | HM  n = 105 | HL  n = 127 | LH  n = 80 | LM  n = 97 | LL  n = 92 | p – Values ^a^ | p_adjust_ -Values ^b^ |
| --- | --- | --- | --- | --- | --- | --- | --- | --- | --- |
| Sex, n (%) |  |  |  |  |  |  |  | 0.99 | 0.99 |
| Women | 394 (63) | 77 (61) | 64 (61) | 81 (64) | 52 (65) | 62 (64) | 58 (63) |  |  |
| Men | 233 (37) | 49 (39) | 41 (39) | 46 (36) | 28 (35) | 35 (36) | 34 (37) |  |  |
| Age, y | 52.0  (43.0, 62.5) | 45.0  (40.0, 53.0) | 52.0  (43.0, 63.0) | 61.0  (52.0, 67.5) | 48.0  (42.0, 54.0) | 51.0  (42.0, 62.0) | 59.0  (50.0, 68.3) | <0.001 | <0.001 |
| Age group, n (%) |  |  |  |  |  |  |  | <0.001^†^ | <0.001^†^ |
| Younger adults,  < 65 years | 497 (79) | 120 (95) | 83 (79) | 78 (61) | 77 (96) | 81 (84) | 58 (63) |  |  |
| Older adults,  ≥ 65 years | 130 (21) | 6 (4.8) | 22 (21) | 49 (39) | 3 (3.8) | 16 (16) | 34 (37) |  |  |
| Height, cm | 172.0  (166.0, 180.0) | 175.0  (169.0, 183.0) | 171.0  (168.0, 178.0) | 169.0  (162.0, 176.0) | 175.0  (170.0, 185.0) | 170.0  (165.0, 179.0) | 168.3  (163.0, 175.0) | <0.001 | <0.001 |
| Weight, kg | 95.7  (78.6, 122.1) | 98.7  (85.7, 128.7) | 95.2  (79.9, 107.6) | 80.9  (68.2, 92.2) | 129.0  (101.3, 155.1) | 98.5  (78.3, 136.4) | 92.1  (74.3, 118.4) | <0.001 | <0.001 |
| BMI, kg/m² | 31.3  (26.4, 41.4) | 31.8  (27.3, 41.9) | 31.0  (26.5, 37.4) | 27.6  (24.1, 31.5) | 43.9  (30.9, 48.2) | 34.1  (26.8, 45.9) | 31.9  (26.6, 40.7) | <0.001 | <0.001 |
| BMI class |  |  |  |  |  |  |  | <0.001^†^ | <0.001^†^ |
| Normal weight | 119 (19) | 22 (17) | 17 (16) | 42 (33) | 3 (3.8) | 17 (18) | 18 (20) |  |  |
| Overweight | 154 (25) | 28 (22) | 30 (29) | 45 (35) | 11 (14) | 21 (22) | 19 (21) |  |  |
| Obesity class I | 107 (17) | 27 (21) | 26 (25) | 14 (11) | 12 (15) | 13 (13) | 15 (16) |  |  |
| Obesity class II | 67 (11) | 12 (9.5) | 11 (10) | 15 (12) | 7 (8.8) | 10 (10) | 12 (13) |  |  |
| Obesity class III | 180 (29) | 37 (29) | 21 (20) | 11 (8.7) | 47 (59) | 36 (37) | 28 (30) |  |  |
| Glucose, mg/dL | 98.0  (90.0,108.0) | 94.0  (87.3, 103.0) | 96.0  (90.0, 107.0) | 98.0  (90.0, 104.0) | 101.0  (94.0, 108.3) | 102.0  (91.0, 116.0) | 101.5  (90.8, 118.0) | <0.001 | <0.001 |
| Insulin, mlU/L | 12.60  (8.15, 21.10) | 10.95  (7.65, 19.05) | 10.50  (7.60, 16.30) | 11.10  (7.35, 17.65) | 17.45  (12.25, 28.12) | 15.50  (9.50, 27.80) | 13.45  (9.15, 30.55) | <0.001 | <0.001 |
| HOMA-IR | 3.08  (1.92, 5.64) | 2.67  (1.78, 4.72) | 2.54  (1.72, 4.16) | 2.64  (1.78, 4.31) | 4.46  (2.81, 7.51) | 4.18  (2.05, 7.59) | 3.82  (2.16, 8.23) | <0.001 | <0.001 |
| **Variable** | **Overall**  **n = 627** | **HH**  **n = 126** | **HM**  **n = 105** | **HL**  **n = 127** | **LH**  **n = 80** | **LM**  **n = 97** | **LL**  **n = 92** | **p – Values ^a^** | **p_adjust_ -Values ^b^** |
| Triglycerides, mg/dL | 119.0  (84.0, 166.0) | 111.5  (78.0, 143.8) | 104.0  (76.0, 165.0) | 116.0  (80.5, 160.0) | 127.5  (99.0, 160.0) | 139.0  (103.0, 193.0) | 124.5  (92.0, 181.3) | <0.001 | <0.001 |
| CRP, mg/L | 3.00  (1.00, 7.00) | 3.00  (1.00, 6.00) | 2.00  (1.00, 5.00) | 2.00  (1.00, 5.00) | 5.00  (2.00, 9.00) | 3.00  (1.00, 9.00) | 3.50  (1.75, 8.25) | 0.002 | 0.002 |
| IL-6, pg/mL | 4.00  (2.80, 5.70) | 3.95  (2.62, 5.20) | 3.70  (2.60, 5.30) | 3.60  (2.65, 5.35) | 4.60  (3.10, 6.20) | 3.80  (3.00, 6.00) | 5.15  (3.48, 7.12) | <0.001 | <0.001 |
| BP systolic, mmHg | 130.0  (120.0, 140.0) | 130.0  (120.0, 140.0) | 130.0  (120.0, 140.0) | 130.0  (120.0, 140.0) | 140.0  (130.0, 140.0) | 135.0  (130.0, 140.0) | 135.0  (128.8, 140.0) | <0.001 | <0.001 |
| BP diastolic, mmHg | 80.0  (80.0, 90.0) | 80.0  (80.0, 88.8) | 80.0  (80.0, 85.0) | 80.0  (80.0, 80.0) | 80.0  (80.0, 90.0) | 80.0  (80.0, 90.0) | 80.0  (80.0, 85.0) | 0.005 | 0.005 |
| Handgrip strength, kg | 28.77  (23.08, 39.00) | 33.28  (29.68, 48.23) | 26.63  (24.93, 41.50) | 21.30  (18.75, 30.15) | 33.47  (29.88, 50.64) | 26.23  (24.40, 42.40) | 20.54  (17.89, 29.28) | <0.001 | <0.001 |
| Handgrip strength /body weight, kg/kg | 0.30  (0.23, 0.40) | 0.36  (0.28, 0.50) | 0.33  (0.25, 0.43) | 0.28  (0.23, 0.37) | 0.28  (0.23, 0.37) | 0.31  (0.23, 0.40) | 0.25  (0.16, 0.32) | <0.001 | <0.001 |

Abbreviations: adjust= adjusted; BMI= body mass index; HH= high sports activity and high handgrip strength; HM= high sports activity and medium handgrip strength; HL= high sports activity and low handgrip strength; LH= low sports activity and high handgrip strength; LM= low sports activity and medium handgrip strength; LL= low sports activity and low handgrip strength.

^a, b^ P values were derived from Kruskal-Wallis test unless otherwise indicated and were adjusted for multiple testing using False discovery rate correction.

* Values are shown as median (interquartile range) or n (%).

^†^From Pearson's Chi-squared test.

**Table S3 Group comparisons of health status including diagnosed diseases of interest (n = 627)***

| Variable | Overall  n = 627 | HH  n = 126 | HM  n = 105 | HL  n = 127 | LH  N = 80 | LM  n = 97 | LL  n = 92 | p – Values ^a^ | p_adjust_ -Values ^b^ |  |
| --- | --- | --- | --- | --- | --- | --- | --- | --- | --- | --- |
| Medication regularly, n (%) | 470 (75) | 81 (64) | 72 (69) | 94 (74) | 64 (80) | 79 (81) | 80 (87) | 0.001 | 0.005 |  |
| Diabetes, n (%) | 111 (18) | 18 (14) | 17 (16) | 15 (12) | 15 (19) | 23 (24) | 23 (25) | 0.074 | 0.17 |  |
| Hypertension, n (%) | 312 (50) | 48 (38) | 45 (43) | 54 (43) | 57 (71) | 55 (57) | 53 (58) | <0.001 | <0.001 |  |
| CHD, n (%) | 24 (3.8) | 2 (1.6) | 4 (3.8) | 5 (3.9) | 2 (2.5) | 4 (4.1) | 7 (7.6) | 0.37 | 0.56 |  |
| Neuropathy, n (%) | 93 (15) | 15 (12) | 19 (18) | 19 (15) | 10 (12) | 15 (15) | 15 (16) | 0.81 | 0.83 |  |
| Rheumatoid arthritis, n (%) | 52 (8.3) | 5 (4.0) | 4 (3.8) | 13 (10) | 6 (7.5) | 9 (9.3) | 15 (16) | 0.013 | 0.039 |  |
| Liver disease, n (%) | 36 (5.7) | 7 (5.6) | 3 (2.9) | 9 (7.1) | 4 (5.0) | 6 (6.2) | 7 (7.6) | 0.72 | 0.83 |  |
| IBD, n (%) | 41 (6.5) | 6 (4.8) | 5 (4.8) | 10 (7.9) | 6 (7.5) | 8 (8.2) | 6 (6.5) | 0.83 | 0.83 |  |
| IBS, n (%) | 21 (3.3) | 1 (0.8) | 7 (6.7) | 6 (4.7) | 2 (2.5) | 3 (3.1) | 2 (2.2) | 0.19 | 0.35 |  |
| Abbreviations: CHD = coronary heart disease, IBD = inflammatory bowel disease, IBS = irritable bowel syndrome, HH = high sports activity and high handgrip strength, HM = high sports activity and medium handgrip strength, HL = high sports activity and low handgrip strength, LH = low sports activity and high handgrip strength, LM = low sports activity and medium handgrip strength, LL = low sports activity and low handgrip strength.  ^a, b^ P values were derived from Pearson's Chi-squared test and were adjusted for multiple testing using False discovery rate correction.  * Values are shown as n (%). | | | | | | | | | | |

**Table S4 Group comparisons regarding socioeconomic status (SES), lifestyle and activities (n = 627)***

| Variable | n | Overall  n = 627 | HH  n = 126 | HM  n = 105 | HL  n = 127 | LH  n = 80 | LM  n = 97 | LL  n = 92 | p – Values ^a^ | p_adjust_ -Values ^b^ |
| --- | --- | --- | --- | --- | --- | --- | --- | --- | --- | --- |
| **SES** |  |  |  |  |  |  |  |  |  |  |
| **School graduation,**  **n (%)** | 627 |  |  |  |  |  |  |  | 0.007 | 0.013 |
| No degree |  | 7 (1.1) | 0 (0) | 0 (0) | 1 (0.8) | 1 (1.2) | 1 (1.0) | 4 (4.3) |  |  |
| Low degree |  | 173 (28) | 28 (22) | 26 (25) | 26 (20) | 26 (32) | 28 (29) | 39 (42) |  |  |
| Intermediate degree |  | 226 (36) | 46 (37) | 40 (38) | 54 (43) | 21 (26) | 35 (36) | 30 (33) |  |  |
| High degree |  | 74 (12) | 20 (16) | 10 (9.5) | 17 (13) | 13 (16) | 8 (8.2) | 6 (6.5) |  |  |
| University qualified |  | 147 (23) | 32 (25) | 29 (28) | 29 (23) | 19 (24) | 25 (26) | 13 (14) |  |  |
| **Vocational graduation, n (%)** | 627 |  |  |  |  |  |  |  | 0.17 | 0.25 |
| None |  | 43 (6.9) | 2 (1.6) | 5 (4.8) | 9 (7.1) | 9 (11) | 5 (5.2) | 13 (14) |  |  |
| In training |  | 11 (1.8) | 2 (1.6) | 2 (1.9) | 3 (2.4) | 1 (1.2) | 1 (1.0) | 2 (2.2) |  |  |
| Vocational training |  | 311 (50) | 65 (52) | 46 (44) | 54 (43) | 42 (52) | 52 (54) | 52 (57) |  |  |
| Vocational degree |  | 106 (17) | 23 (18) | 21 (20) | 23 (18) | 13 (16) | 14 (14) | 12 (13) |  |  |
| College |  | 64 (10) | 13 (10) | 14 (13) | 13 (10) | 7 (8.8) | 10 (10) | 7 (7.6) |  |  |
| University |  | 92 (15) | 21 (17) | 17 (16) | 25 (20) | 8 (10) | 15 (15) | 6 (6.5) |  |  |
| **Employment** | 627 |  |  |  |  |  |  |  | <0.001 | <0.001 |
| Full time |  | 215 (34) | 70 (56) | 36 (34) | 29 (23) | 31 (39) | 34 (35) | 15 (16) |  |  |
| Part time |  | 99 (16) | 28 (22) | 20 (19) | 13 (10) | 18 (22) | 12 (12) | 8 (8.7) |  |  |
| Pensioner |  | 182 (29) | 7 (5.6) | 27 (26) | 64 (50) | 10 (12) | 28 (29) | 46 (50) |  |  |
| Unemployed |  | 46 (7.3) | 9 (7.1) | 6 (5.7) | 3 (2.4) | 8 (10) | 10 (10) | 10 (11) |  |  |
| Other |  | 67 (11) | 10 (7.9) | 14 (13) | 16 (13) | 6 (7.5) | 11 (11) | 10 (11) |  |  |
| > 1 employment |  | 18 (2.9) | 2 (1.6) | 2 (1.9) | 2 (1.6) | 7 (8.8) | 2 (2.1) | 3 (3.3) |  |  |
| **Cohabitation** | 627 |  |  |  |  |  |  |  | 0.27 | 0.37 |
| Single household |  | 120 (19) | 23 (18) | 16 (15) | 24 (19) | 11 (14) | 20 (21) | 26 (28) |  |  |
| Living together |  | 469 (75) | 97 (77) | 79 (75) | 94 (74) | 63 (79) | 72 (74) | 64 (70) |  |  |
| Other |  | 38 (6.1) | 6 (4.8) | 10 (9.5) | 9 (7.1) | 6 (7.5) | 5 (5.2) | 2 (2.2) |  |  |
|  |  |  |  |  |  |  |  |  |  |  |
|  |  |  |  |  |  |  |  |  |  |  |
|  |  |  |  |  |  |  |  |  |  |  |
| **Variable** | **n** | **Overall**  **n = 627** | **HH**  **n = 126** | **HM**  **n = 105** | **HL**  **n = 127** | **LH**  **n = 80** | **LM**  **n = 97** | **LL**  **n = 92** | **p – Values ^a^** | **p_adjust_ -Values ^b^** |
| **Content life, n (%)** | 627 |  |  |  |  |  |  |  | <0.001 | <0.001 |
| Very content |  | 115 (18) | 27 (21) | 21 (20) | 36 (28) | 6 (7.5) | 12 (12) | 13 (14) |  |  |
| Content |  | 341 (54) | 63 (50) | 62 (59) | 74 (58) | 43 (54) | 47 (48) | 52 (57) |  |  |
| Not so content |  | 135 (22) | 35 (28) | 17 (16) | 14 (11) | 24 (30) | 29 (30) | 16 (17) |  |  |
| Not at all content |  | 36 (5.7) | 1 (0.8) | 5 (4.8) | 3 (2.4) | 7 (8.8) | 9 (9.3) | 11 (12) |  |  |
| **Lifestyle & activities** |  |  |  |  |  |  |  |  |  |  |
| Smoker, n (%) | 627 | 124 (20) | 20 (16) | 23 (22) | 20 (16) | 20 (25) | 20 (21) | 21 (23) | 0.44 | 0.51 |
| Smoking habit, n (%) | 627 |  |  |  |  |  |  |  | 0.67 | 0.72 |
| Never smoked |  | 196 (31) | 43 (34) | 38 (36) | 35 (28) | 19 (24) | 31 (32) | 30 (33) |  |  |
| Smoked < 3 months |  | 50 (8.0) | 13 (10) | 8 (7.6) | 10 (7.9) | 6 (7.5) | 8 (8.2) | 5 (5.4) |  |  |
| Smoked in the past |  | 257 (41) | 50 (40) | 36 (34) | 62 (49) | 35 (44) | 38 (39) | 36 (39) |  |  |
| Currently smoking |  | 124 (20) | 20 (16) | 23 (22) | 20 (16) | 20 (25) | 20 (21) | 21 (23) |  |  |
| Sleep at day, h/day | 627 | 0.0  (0.0, 1.0) | 0.0  (0.0, 0.2) | 0.0  (0.0, 0.5) | 0.2  (0.0, 0.8) | 0.0  (0.0, 0.5) | 0.5  (0.0, 1.0) | 0.1  (0.0, 1.0) | <0.001 | <0.001 |
| Sleep at night, h/night | 627 | 7.0  (6.0, 8.0) | 7.0  (6.0, 8.0) | 7.0  (6.0, 8.0) | 7.0  (6.5, 8.0) | 7.0  (6.0, 7.2) | 7.0  (6.0, 8.0) | 7.0  (6.0, 8.0) | 0.037 | 0.061 |
| Daily activity, h/week | 627 | 15.2  (8.9, 25.5) | 17.6  (9.0, 23.4) | 17.0  (10.8, 25.0) | 17.0  (8.6, 27.0) | 15.0  (9.7, 28.0) | 13.5  (7.1, 22.0) | 14.8  (8.4, 23.1) | 0.34 | 0.42 |
| Adapted dietary inflammatory index | 590 | 0.6  (-1.6, 2.6) | 0.6  (-1.6, 2.7) | 0.0  (-1.7, 1.5) | 0.6  (-1.9, 2.2) | 1.3  (-0.8, 3.9) | 0.7  (-1.7, 2.6) | 1.1  (-1.4, 3.1) | 0.016 | 0.049 |
| Tryptophan intake, g/day | 590 | 6.0  (5.8, 6.3) | 6.0  (5.8, 6.2) | 6.1  (5.9, 6.3) | 6.1  (5.8, 6.3) | 6.0  (5.6, 6.2) | 6.1  (5.7, 6.2) | 6.0  (5.8, 6.2) | 0.34 | 0.50 |
| Healthy lifestyle score | 588 |  |  |  |  |  |  |  | <0.001 | <0.001 |
| 1 |  | 36 (6.1) | 2 (1.7) | 1 (0.9) | 5 (5.0) | 12 (16) | 8 (9.4) | 8 (8.7) |  |  |
| 2 |  | 173 (29) | 14 (12) | 9 (7.8) | 16 (16) | 42 (57) | 44 (52) | 48 (52) |  |  |
| 3 |  | 221 (38) | 53 (44) | 46 (40) | 40 (40) | 19 (26) | 27 (32) | 36 (39) |  |  |
| 4 |  | 135 (23) | 45 (37) | 46 (40) | 37 (37) | 1 (1.4) | 6 (7.1) | 0 (0) |  |  |
| 5 |  | 23 (3.9) | 7 (5.8) | 14 (12) | 2 (2.0) | 0 (0) | 0 (0) | 0 (0) |  |  |

*Note:* h=hours, SES=socioeconomic status, adj.= adjusted; HH= high sports activity and high handgrip strength; HM= high sports activity and medium handgrip strength; HL= high sports activity and low handgrip strength; LH= low sports activity and high handgrip strength; LM= low sports activity and medium handgrip strength; LL= low sports activity and low handgrip strength.

^a, b^ P values were derived from Kruskal-Wallis rank sum test unless otherwise indicated and were adjusted for multiple testing using False discovery rate correction.

* Values are shown as median (interquartile range) or n (%).

†From Pearson's Chi-squared test.

**Table S5 Results of the enrichment analysis using The Human Metabolome Database (HMDB) results from the MetaboAnalyst analysis stratified by sex and bio sample type**

| **enriched pathway** | **total** | **hits** | **raw p – Values ^a^** | **Holm p – Values ^b^** | **FDR p – Values ^c^** |
| --- | --- | --- | --- | --- | --- |
| **Serum samples** |  |  |  |  |  |
| *Women* |  |  |  |  |  |
| Sulfate/Sulfite metabolism | 22 | 1 | 0.0435 | 1.0000 | 1.0000 |
| Histidine metabolism | 42 | 1 | 0.0821 | 1.0000 | 1.0000 |
| **Urine samples** |  |  |  |  |  |
| *Women* |  |  |  |  |  |
| Caffeine metabolism | 23 | 3 | 0.0004 | 0.0342 | 0.0342 |
| Methyl-histidine metabolism | 4 | 1 | 0.0277 | 1.0000 | 0.9390 |
| Sphingolipid metabolism | 40 | 2 | 0.0287 | 1.0000 | 0.9390 |
| Phosphatidylethanolamine biosynthesis | 12 | 1 | 0.0811 | 1.0000 | 1.0000 |
| Phosphatidylcholine biosynthesis | 14 | 1 | 0.0941 | 1.0000 | 1.0000 |
| Phospholipid biosynthesis | 29 | 1 | 0.1860 | 1.0000 | 1.0000 |
| Ammonia recycling | 31 | 1 | 0.1980 | 1.0000 | 1.0000 |
| Beta-Alanine metabolism | 34 | 1 | 0.2150 | 1.0000 | 1.0000 |
| Histidine metabolism | 42 | 1 | 0.2600 | 1.0000 | 1.0000 |
| *Men* |  |  |  |  |  |
| Pantothenate and CoA biosynthesis | 21 | 1 | 0.0210 | 1.0000 | 1.0000 |
| Beta-Alanine metabolism | 34 | 1 | 0.0393 | 1.0000 | 1.0000 |

*Note:* total = total number of compounds in the pathway; hits = actually matched number from the user-uploaded data.

^a^ P = original P - Values calculated from the enrichment analysis (raw); ^b^ P = P - Values adjusted by Holm-Bonferroni method (Holm); ^c^ P = P - Values adjusted using False Discovery Rate (FDR)

**Table S6** **Odds ratios (OR) and 95% Confidence intervals (95% CI; P-value) of potential predictors for a low physical fitness (LL) from multivariate binomial logistic regression model with the dependent group high physical fitness (HH) (n = 207)**

| **Dependent: HH** |  | **HH** | **LL** | **OR (univariable)** | **OR (multivariable)** |
| --- | --- | --- | --- | --- | --- |
| Sex, n (%) | Women (reference) | 74 (57.4) | 55 (42.6) | - | - |
|  | Men | 48 (61.5) | 30 (38.5) | 0.84 (0.47-1.49, p =0 .554) | 0.45 (0.18-1.09, p = 0.081) |
| Age, y | Mean (SD) | 45.9 (11.0) | 58.7 (13.0) | 1.09 (1.06-1.13, p < 0.001) | 1.12 (1.08-1.17, p < 0.001) |
| CRP, mg/L | Mean (SD) | 5.0 (5.7) | 6.4 (7.9) | 1.03 (0.99-1.08, p = 0.131) | 1.09 (1.00-1.18, p = 0.058) |
| HOMA | Mean (SD) | 4.1 (4.6) | 6.6 (8.8) | 1.07 (1.02-1.13, p = 0.019) | 1.06 (0.98-1.16, p = 0.208) |
| IL-6, pg/mL | Mean (SD) | 4.5 (3.0) | 8.1 (21.7) | 1.13 (1.03-1.26, p = 0.017) | 1.00 (0.96-1.14, p = 0.983) |
| Triglycerides, mg/dL | Mean (SD) | 118.8 (60.0) | 150.7 (97.5) | 1.01 (1.00-1.01, p = 0.007) | 1.00 (1.00-1.01, p = 0.543) |
| Medication | no (reference) | 45 (80.4) | 11 (19.6) | - | - |
|  | yes | 77 (51.0) | 74 (49.0) | 3.93 (1.95-8.53, p < 0.001) | 1.66 (0.57-5.15, p = 0.363) |
| Sleep at day, h/day | Mean (SD) | 0.2 (0.6) | 0.6 (1.2) | 1.96 (1.29-3.27, p = 0.005) | 2.72 (1.51-5.42, p = 0.002) |
| Caffeine intake, mg/day | Mean (SD) | 0.0 (1.1) | 0.1 (1.0) | 1.03 (0.79-1.33, p = 0.839) | 1.18 (0.77-1.82, p = 0.450) |
| Tryptophan intake, g/day | Mean (SD) | 6.0 (0.4) | 6.0 (0.4) | 0.65 (0.32-1.30, p = 0.228) | 0.78 (0.24-2.35, p = 0.665) |
| Healthy lifestyle score | Mean (SD) | 3.3 (0.8) | 2.4 (0.8) | 0.22 (0.14-0.34, p < 0.001) | 0.18 (0.09-0.33, p < 0.001) |
| *Note:* HH = high sports activity and high handgrip strength, LL = low sports activity and low handgrip strength, SD = Standard deviation.  Number in data frame = 207, Number in model = 206, Missing = 1, AIC = 167.1, C-statistic = 0.92, H&L = Chi-sq (8) 8.11 (p = .422) | | | | | |

**Table S7 Odds ratios (OR) and 95% Confidence intervals (95% CI; P-value) of potential predictors for a low physical fitness (LL) from multivariate binomial logistic regression model with the dependent group high physical fitness (HH) (n = 203) including microbiota diversity**

| **Dependent: HH** |  | **HH** | **LL** | **OR (univariable)** | **OR (multivariable)** |
| --- | --- | --- | --- | --- | --- |
| Sex | Women (reference) | 72 (57.1) | 54 (42.9) | - | - |
|  | Men | 48 (62.3) | 29 (37.7) | 0.81 (0.45-1.44, p = .465) | 0.43 (0.17-1.04, p = .067) |
| Age, y | Mean (SD) | 45.9 (10.8) | 58.7 (13.0) | 1.10 (1.07-1.13, p < .001) | 1.12 (1.08-1.18, p < .001) |
| CRP, mg/L | Mean (SD) | 5.0 (5.8) | 6.4 (7.9) | 1.03 (0.99-1.08, p = .162) | 1.08 (0.99-1.18, p = .098) |
| HOMA | Mean (SD) | 4.1 (4.7) | 6.6 (8.9) | 1.07 (1.02-1.13, p = .018) | 1.06 (0.98-1.17, p = .180) |
| IL 6, pg/mL | Mean (SD) | 4.5 (3.0) | 8.3 (21.9) | 1.14 (1.03-1.27, p = .014) | 1.01 (0.96-1.15, p = .920) |
| Triglycerides, mg/dL | Mean (SD) | 118.2 (60.1) | 149.8 (97.8) | 1.01 (1.00-1.01, p = .009) | 1.00 (1.00-1.01, p = .636) |
| Medication | no (reference) | 44 (80.0) | 11 (20.0) | - | - |
|  | yes | 76 (51.4) | 72 (48.6) | 3.79 (1.87-8.24, p < .001) | 1.57 (0.53-4.92, p = .427) |
| Sleep at day, h/day | Mean (SD) | 0.2 (0.6) | 0.7 (1.2) | 2.01 (1.31-3.37, p = .004) | 2.79 (1.52-5.74, p = .002) |
| Healthy lifestyle score | Mean (SD) | 3.3 (0.8) | 2.4 (0.8) | 0.23 (0.14-0.35, p < .001) | 0.19 (0.10-0.33, p < .001) |
| Caffeine intake, mg/day | Mean (SD) | 0.0 (1.1) | 0.1 (1.0) | 1.03 (0.80-1.34, p = .798) | 1.18 (0.76-1.82, p = .456) |
| Tryptophan intake, g/day | Mean (SD) | 6.0 (0.4) | 6.0 (0.4) | 0.65 (0.32-1.30, p = .227) | 0.80 (0.24-2.43, p = .698) |
| Chao1 | Mean (SD) | 233.2 (67.8) | 227.9 (74.3) | 1.00 (0.99-1.00, p = .603) | 1.00 (0.99-1.01, p = .857) |
| InvSimpson | Mean (SD) | 31.0 (19.1) | 27.2 (13.8) | 0.99 (0.97-1.00, p = .125) | 1.00 (0.97-1.03, p = .828) |
| *Note:* HH = high sports activity and high handgrip strength, LL = low sports activity and low handgrip strength, SD = Standard deviation.  Number in data frame = 203, Number in model = 203, Missing = 0, AIC = 169.8, C-statistic = 0.92, H&L = Chi-sq (8) 8.19 (p = .415) | | | | | |
