# Supplementary material for: Healthy lifestyle, daytime sleepiness, and gut microbiome composition are determinants of functional strength in humans: a cross-sectional study
Source: Sci Rep. 2025 May 19;15:17378. doi: 10.1038/s41598-025-02519-5 (PMC12089321; doi:10.1038/s41598-025-02519-5)
Supplement: Supplementary file 3 — Supplementary Material 3 [file 41598_2025_2519_MOESM3_ESM.docx]

**Healthy lifestyle, daytime sleepiness, and gut microbiome composition are determinants of functional strength in humans: a cross-sectional study**

Friederike Norkeweit^1^, Kristina Schlicht^1^, Nathalie Rohmann^1^, Katharina Hartmann^1^, Kathrin Türk^1^, Ute Settgast^2^, Dominik M. Schulte^1,2^, Felix Gilbert^3^, Tobias Demetrowitsch^4^, Fynn Brix^4^, Corinna Bang^3^, Andre Franke^3^, Karin Schwarz^4^, Matthias Laudes^1,2^ and Corinna Geisler^1,^*

^1^Institute of Diabetes and Clinical Metabolic Research, University Medical Center Schleswig-Holstein and Kiel University, Kiel 24105, Germany

^2^Division of Endocrinology, Diabetes and Clinical Nutrition, Department of Internal Medicine I, University Medical Center Schleswig-Holstein, Campus Kiel, Kiel 24105, Germany

^3^Institute of Clinical Molecular Biology (IKMB), Kiel University, Kiel 24118, Germany

^4^Division of Food Technology, Institute of Human Nutrition and Food Science, Kiel University, Kiel 24105, Germany

*Address correspondence to: Corinna Geisler, PhD, PD; Institute of Diabetes and Clinical Metabolic Research, University Medical Center, Schleswig-Holstein and Kiel University, Kiel 24105, Germany. E-mail: corinna.geisler@uksh.de; Telephone: +4943150022446


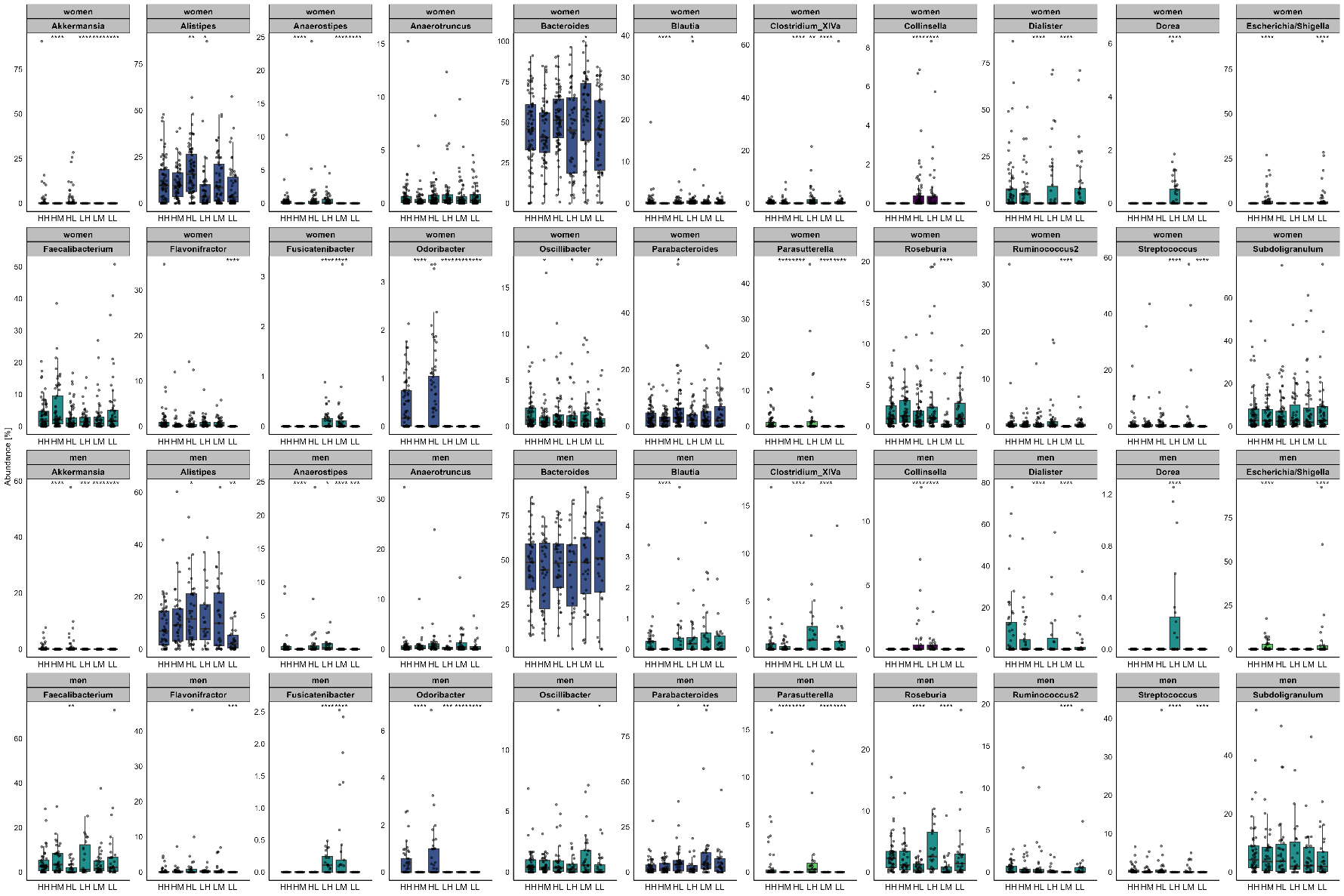
**Fig. S1** Relative abundance of taxa at the genus level with group differences related to the six functional strength groups (n = 578)

*Abbreviations:* HH= high sports activity and high handgrip strength; HM= high sports activity and medium handgrip strength; HL= high sports activity and low handgrip strength; LH= low sports activity and high handgrip strength; LM= low sports activity and medium handgrip strength; LL= low sports activity and low handgrip strength.

p-values were derived from the Mann-Whitney-U rank sum test with comparison to the reference group HH (significance: * p ≤ .05, ** P ≤ .01, *** P ≤ .001, **** P ≤ .0001). Colors are related to the corresponding phyla: purple = Actinobacteria, blue = Bacteroidetes, dark green = Firmicutes, light green = Proteobacteria, and yellow = Verrucomicrobia.

**
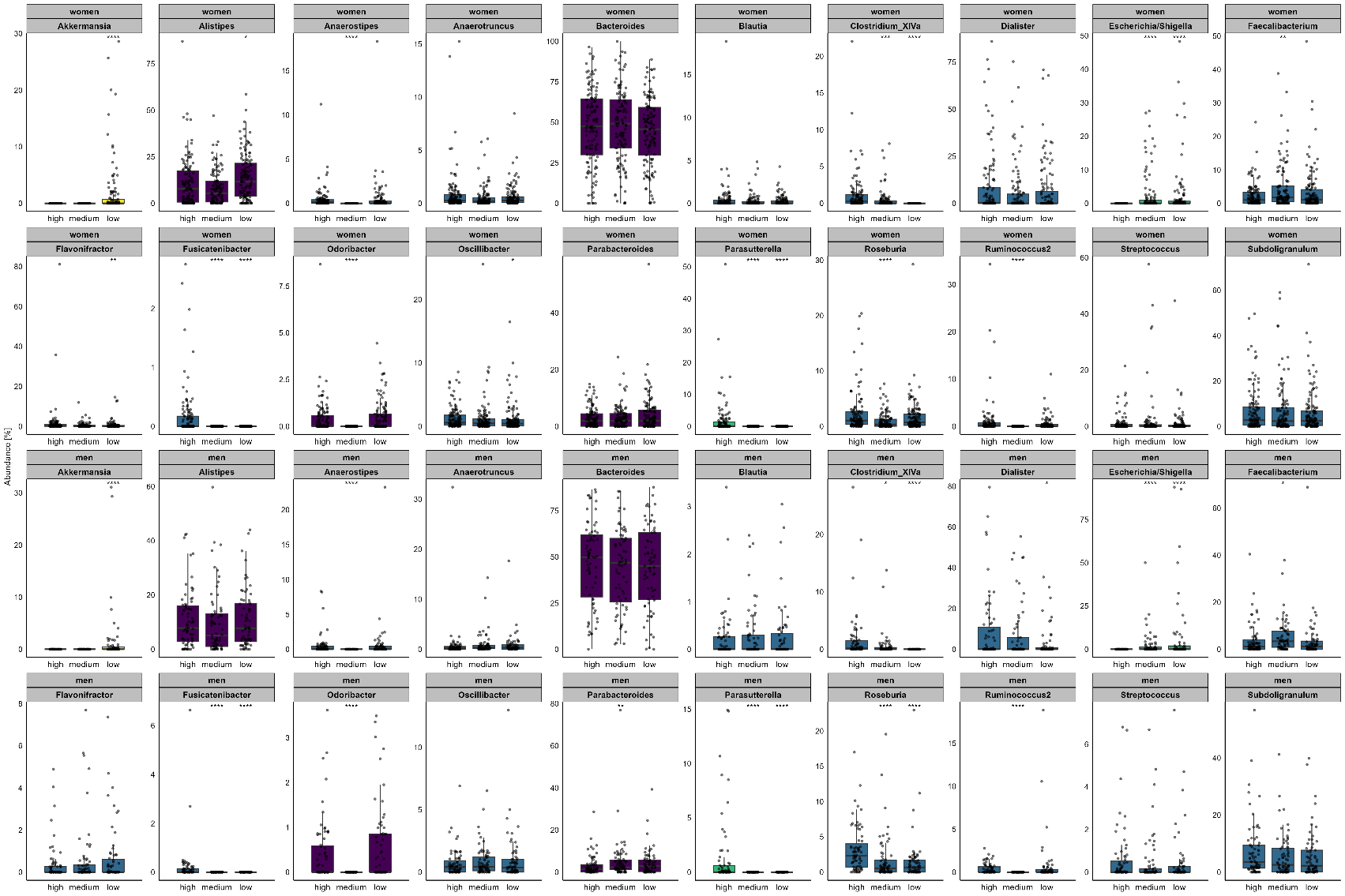
****Fig. S2** Relative abundance of taxa at the genus level with group differences related to three handgrip strength (HGS) groups (n = 578)

*Abbreviations:* high = high handgrip strength; medium = medium handgrip strength; low = low handgrip strength.

p-values were derived from the Mann-Whitney-U rank sum test with comparison to the reference group high (significance: * p ≤ .05, ** P ≤ .01, *** P ≤ .001, **** P ≤ .0001). Colors are related to the corresponding phyla: purple = Bacteroidetes, blue= Firmicutes, light green = Proteobacteria, and yellow = Verrucomicrobia.

**
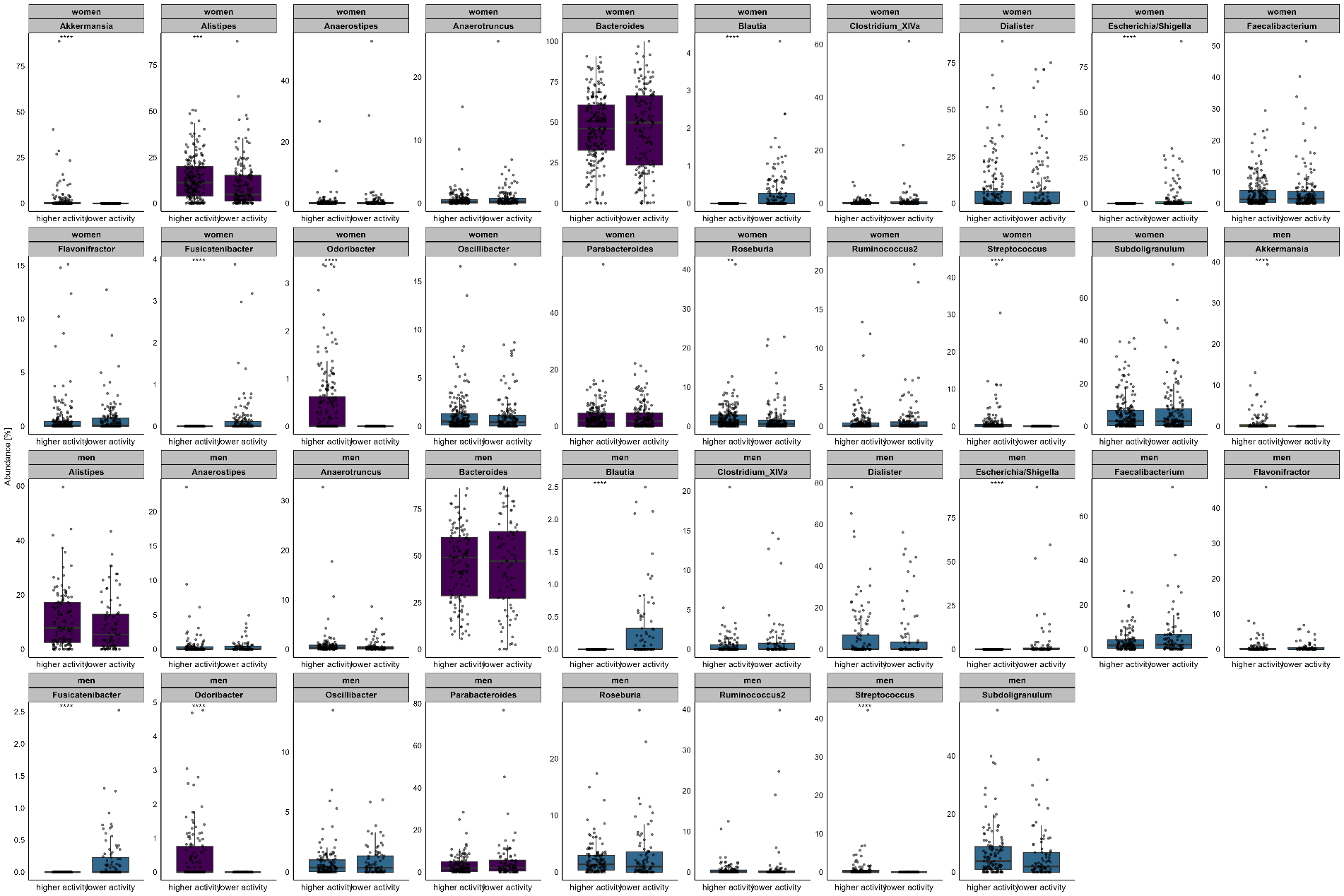
Fig. S3** Relative abundance of taxa at the genus level with group differences related to the two activity groups (n = 578)

p - values were derived from the Mann-Whitney-U rank sum test (significance: * p ≤ .05, ** P ≤ .01, *** P ≤ .001, **** P ≤ .0001). Colors are related to the corresponding phyla: purple = Bacteroidetes, blue= Firmicutes, light green = Proteobacteria, and yellow = Verrucomicrobia.


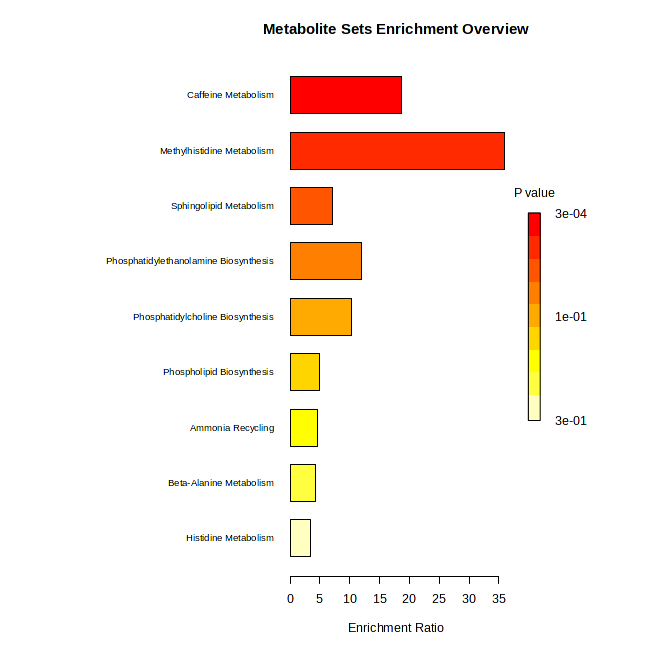


**Fig. S4** Results of fold-enrichment analysis of potential metabolites in the urine of women. The vertical axis represents the pathname and the horizontal axis represents the enrichment ratio using MetaboAnalyst

The p-values are represented by the colors of the bar plots. The smaller the p-value, the closer the color is to red.

.
